# Phosphorylation of the Conserved Transcription Factor ATF-7 by PMK-1 p38 MAPK Regulates Innate Immunity in *Caenorhabditis elegans*

**DOI:** 10.1371/journal.pgen.1000892

**Published:** 2010-04-01

**Authors:** Robert P. Shivers, Daniel J. Pagano, Tristan Kooistra, Claire E. Richardson, Kirthi C. Reddy, Janelle K. Whitney, Odile Kamanzi, Kunihiro Matsumoto, Naoki Hisamoto, Dennis H. Kim

**Affiliations:** 1 Department of Biology, Massachusetts Institute of Technology, Cambridge, Massachusetts, United States of America; 2 Department of Molecular Biology, Nagoya University, Nagoya, Japan; University of California San Francisco, United States of America

## Abstract

Innate immunity in *Caenorhabditis elegans* requires a conserved PMK-1 p38 mitogen-activated protein kinase (MAPK) pathway that regulates the basal and pathogen-induced expression of immune effectors. The mechanisms by which PMK-1 p38 MAPK regulates the transcriptional activation of the *C. elegans* immune response have not been identified. Furthermore, in mammalian systems the genetic analysis of physiological targets of p38 MAPK in immunity has been limited. Here, we show that *C. elegans* ATF-7, a member of the conserved cyclic AMP–responsive element binding (CREB)/activating transcription factor (ATF) family of basic-region leucine zipper (bZIP) transcription factors and an ortholog of mammalian ATF2/ATF7, has a pivotal role in the regulation of PMK-1–mediated innate immunity. Genetic analysis of loss-of-function alleles and a gain-of-function allele of *atf-7*, combined with expression analysis of PMK-1–regulated genes and biochemical characterization of the interaction between ATF-7 and PMK-1, suggest that ATF-7 functions as a repressor of PMK-1–regulated genes that undergoes a switch to an activator upon phosphorylation by PMK-1. Whereas loss-of-function mutations in *atf-7* can restore basal expression of PMK-1–regulated genes observed in the *pmk-1* null mutant, the induction of PMK-1–regulated genes by pathogenic *Pseudomonas aeruginosa* PA14 is abrogated. The switching modes of ATF-7 activity, from repressor to activator in response to activated PMK-1 p38 MAPK, are reminiscent of the mechanism of regulation mediated by the corresponding ancestral Sko1p and Hog1p proteins in the yeast response to osmotic stress. Our data point to the regulation of the ATF2/ATF7/CREB5 family of transcriptional regulators by p38 MAPK as an ancient conserved mechanism for the control of innate immunity in metazoans, and suggest that ATF2/ATF7 may function in a similar manner in the regulation of mammalian innate immunity.

## Author Summary

We have investigated mechanisms of how the soil nematode *Caenorhabditis elegans* interacts with pathogenic bacteria. Previously, we have established that a conserved PMK-1 p38 mitogen-activated protein kinase (MAPK) pathway regulates immunity in *C. elegans*, establishing the conservation of key innate immune signaling pathways of mammals in the immune response of *C. elegans*. Whereas multiple proteins have been identified as potential targets of p38 MAPK in immunity, the identification of physiological substrates of p38 MAPK in mammalian organisms has been challenging. Here, using a forward genetic approach to identify downstream regulators of the *C. elegans* innate immune response, we have characterized the transcription factor ATF-7, a conserved member of the basic-region leucine zipper (bZIP) transcription factor family orthologous to mammalian ATF2. We find that ATF-7 functions as a transcriptional regulator of PMK-1 MAPK–mediated innate immunity, functioning as a repressor of immune gene expression that undergoes a switch to an activator upon activation by PMK-1. Our data point to the regulation of the ATF2/ATF7/CREB5 family of transcriptional regulators by p38 MAPK as an ancient conserved mechanism for the control of innate immunity in metazoans and suggests a mechanism by which the protean effects of p38 MAPK on the mammalian innate immune response may be mediated.

## Introduction

Studies of innate immunity in phylogenetically diverse organisms have revealed the conservation of key signaling pathways mediating pathogen defense [1],[2]. In mammals, the initial encounter between cells of the immune system and pathogenic bacteria triggers the activation of the innate immune response to infection, which is under the control of the transcription factor NF-kB and stress-activated mitogen-activated protein kinases (MAPKs) p38 and JNK [3]. Multiple phosphorylation targets for p38 and JNK MAPKs have been identified in mammalian systems, including members of the cyclic AMP-responsive element binding (CREB)/activating transcription factor (ATF) family such as ATF2 [4], activating protein 1 (AP-1), transcription factors Fos and Jun [5], and multiple kinases including the MAPK-activated protein kinase MK2 [6]. Genetic analysis of MK2 knockout mice is suggestive of a role for p38 MAPK regulation of MK2 in the post-transcriptional regulation of TNF-α production [7]. However, genetic analysis of transcription factor targets of p38 and JNK MAPKs has been limited by lethality of knockouts and possible redundancy [8], and thus the identification and characterization of the physiologically relevant targets of MAPK signaling in innate immunity remains a major challenge [9].

We have focused on the genetic dissection of innate immunity in the nematode *Caenorhabditis elegans*. Previously, we identified a requirement for a conserved NSY-1-SEK-1-PMK-1 MAPK pathway, orthologous to mammalian ASK1 MAPKKK-MKK3/6 MAPKK-p38 MAPK, in *C. elegans* innate immunity [10]. Notably, the loss of PMK-1 p38 MAPK activity in *C. elegans*, unlike the loss of mammalian p38 MAPK, does not affect growth and development of *C. elegans* on non-pathogenic bacteria. The ASK1-MKK3/6-p38 MAPK pathway has been shown to be required for innate immune signaling downstream of Toll-like Receptor-4 (TLR4) in mice [11], whereas NSY-1-SEK-1-PMK-1 signaling in *C. elegans* is TLR-independent and functions downstream of a Toll-Interleukin-1 Receptor (TIR) domain protein TIR-1 [12]–[15], an ortholog of mammalian SARM [16]–[18]. The role of SARM in mammalian innate immunity is somewhat unclear [16],[17], with some studies suggestive of a role for SARM in the inhibition of TRIF-dependent TLR signaling [16]. Recent studies of the PMK-1 pathway are suggestive of a role for protein kinase C-dependent signaling upstream of TIR-1 [19],[20].

The TIR-1-NSY-1-SEK-1-PMK-1 pathway acts cell autonomously in the intestine to regulate innate immunity in *C. elegans* [21], paralleling the role of this pathway in the epidermal response to Drechmeria [15]. The transcriptional profiling of *C. elegans* mutants deficient in PMK-1 pathway activity has identified a number of PMK-1-dependent candidate effector genes, including C-type lectins and putative antimicrobial peptides, many of which are induced by pathogen infection [22]. Whereas the GATA family transcription factor ELT-2 has been implicated in the regulation of *C. elegans* innate immunity in response to intestinal infection [23], in addition to its role in the expression of all intestinally expressed genes [24], the specific targets of PMK-1 p38 MAPK in the regulation of the immune effector response have remained uncertain.

In this paper we report the results of a forward genetic screen for mutants deficient in immune signaling through the PMK-1 p38 MAPK pathway. We report the identification of ATF-7, a putative ortholog of the mammalian ATF2 family of basic-region leucine zipper (bZIP) transcription factors, as a key downstream target of the PMK-1 p38 MAPK pathway in *C. elegans*. Our data establish a pivotal role for ATF-7 as a transcriptional regulator of the PMK-1-mediated innate immune response in *C. elegans*.

## Results

### Isolation and characterization of mutant alleles of *atf-7*

We modified our prior screen for mutants with enhanced susceptibility to pathogens (Esp phenotype) [10] to focus on the identification of genes encoding components of PMK-1 p38 MAPK-dependent innate immunity in *C. elegans*. We used as our starting strain a wild-type (WT) N2-derived strain carrying the *agIs219* transgene, which is comprised of the promoter of a PMK-1-regulated gene, *T24B8.5*, encoding a ShK-like toxin peptide, fused to green fluorescent protein (GFP) and provides an *in vivo* sensor of PMK-1 pathway activity [21]. Mutagenized animals with diminished GFP expression were enriched using the COPAS worm sorter, and this enriched population was subsequently transferred to *Pseudomonas aeruginosa* PA14 for isolation of mutants with diminished PMK-1-dependent reporter expression and an Esp phenotype. From an initial round of high-throughput screening of worms derived from 140 000 mutagenized genomes, we isolated 33 mutants representing five complementation groups using a cutoff for Esp screening that required mutants to be dead prior to the death of any unmutagenized worms treated in parallel (Table 1). Using a combination of complementation testing and sequencing of candidate genes, we determined that four of the complementation groups correspond to genes encoding the established TIR-1-NSY-1-SEK-1-PMK-1 pathway.

**Table 1:** List of isolates from a screen for mutants with diminished GFP expression from the *agIs219* transgene and enhanced susceptibility to killing by *P. aeruginosa* PA14.

Gene	Allele	Mutation
*nsy-1*	*qd1*	Not identified
*nsy-1*	*qd3*	Q256*
*nsy-1*	*qd6*	W391*
*nsy-1*	*qd8*	G440E
*nsy-1*	*qd23*	W514*
*nsy-1*	*qd25*	W157*
*nsy-1*	*qd29*	Q426*
*nsy-1*	*qd31*	P867L
*nsy-1*	*qd38*	G793R
*nsy-1*	*qd41*	451 bp deletion with 2 bp insertion
*nsy-1*	*qd44*	Not identified
*nsy-1*	*qd50*	Splice site
*nsy-1*	*qd52*	Not identified
*nsy-1*	*qd55*	G673E
*sek-1*	*qd12*	R221K
*sek-1*	*qd14*	S103N
*sek-1*	*qd24*	M217I
*sek-1*	*qd26*	E220K
*sek-1*	*qd37*	G199D
*sek-1*	*qd39*	W123*
*sek-1*	*qd60*	G5R
*sek-1*	*qd65*	G212R
*pmk-1*	*qd9*	L296F
*pmk-1*	*qd11*	D161N
*pmk-1*	*qd13*	M209I
*pmk-1*	*qd15*	W353*
*pmk-1*	*qd20*	C173Y
*pmk-1*	*qd47*	W29*
*pmk-1*	*qd49*	Not identified
*tir-1*	*qd2*	A451V
*tir-1*	*qd66*	Q278*
*tir-1*	*qd67*	P460L
*atf-7*	*qd22*	P58S

Mutations in *tir-1* are in reference to gene model *F13B10.1b*. The mutation in *atf-7* is in reference to gene model *C07G2.2a*.

**Figure 1:**
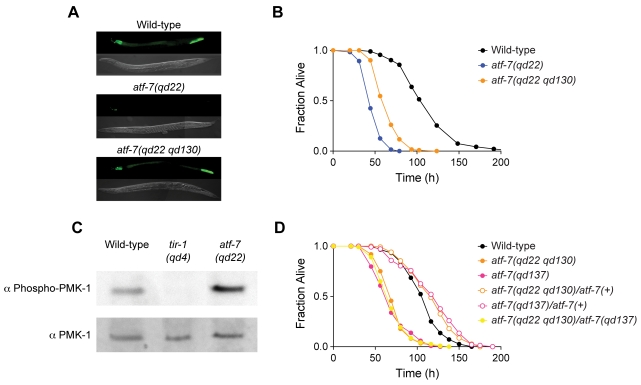
Characterization of *atf-7* mutants that affect signaling downstream of PMK-1 p38 MAPK. (A) Fluorescence microscopy images of GFP expression from the *agIs219* transgene in wild-type, *atf-7(qd22)* and *atf-7(qd22 qd130)* one-day-old adults. (B) Pathogenesis assay of L4 larval stage wild-type worms, *atf-7(qd22)* and *atf-7(qd22 qd130)* mutant animals, on *P. aeruginosa* PA14. All strains carry the *agIs219* transgene. The differences in susceptibility between *atf-7(qd22)* mutant animals and wild-type worms, *atf-7(qd22)* and *atf-7(qd22 qd130)* mutant animals, and *atf-7(qd22 qd130)* mutant animals and wild-type worms are all significant (*p*<0.0001 for each comparison). Replicate data can be seen in Figure S10. (C) Immunoblot analysis of worm lysates from *atf-7(qd22)* worms. Total PMK-1 was identified using a polyclonal antibody generated against *C. elegans* PMK-1. Activated PMK-1 levels were identified using an antibody specific for the doubly phosphorylated TGY motif of activated PMK-1. (D) Pathogenesis assay of L4 larval stage wild-type worms; *atf-7(qd22 qd130)* and *atf-7(qd137)* mutants; and *atf-7(qd22 qd130)/atf-7(+), atf-7(qd137)/atf-7(+)*, and *atf-7(qd22 qd130)/atf-7(qd137)* trans-heterozygotes, on *P. aeruginosa* PA14. All strains carry the *agIs219* transgene. The differences in susceptibility between *atf-7(qd22 qd130)* mutant animals and *atf-7(qd22 qd130)/atf-7(+)* trans-heterozygotes, and *atf-7(qd137)* mutants animals and *atf-7(qd137)/atf-7(+)* trans-heterozygotes are significant (*p*<0.0001 for each comparison). There is no difference in susceptibility between *atf-7(qd22 qd130)* mutant animals and *atf-7(qd22 qd130)/atf-7(qd137)* trans-heterozygotes, and *atf-7(qd137)* mutant animals and *atf-7(qd22 qd130)/atf-7(qd137)* trans-heterozygotes (*p*>0.35 for each comparison). Replicate data can be seen in Figure S13.

The fifth complementation group was defined by a single allele, *qd22*. The *qd22* mutant exhibited a marked decrease in expression of the *agIs219* transgene (Figure 1A) and conferred a strong Esp phenotype (Figure 1B and Figure S10). The lifespan of the *qd22* mutant on relatively non-pathogenic *Escherichia coli* OP50 was comparable to WT (Figure S1 and Figure S16). Using lysates from *qd22* mutant worms, we carried out immunoblotting against the activated form of PMK-1. We found that unlike the other mutants isolated in the screen which carry mutations in PMK-1 pathway components functioning upstream of PMK-1 [21], the *qd22* mutant did not exhibit diminished levels of PMK-1 activation, and in fact had increased levels of activated PMK-1 compared to WT (Figure 1C). Taken together, these data suggested that the *qd22* mutation affected PMK-1-dependent reporter gene expression either at a step downstream of or parallel to PMK-1.

**Figure 2:**
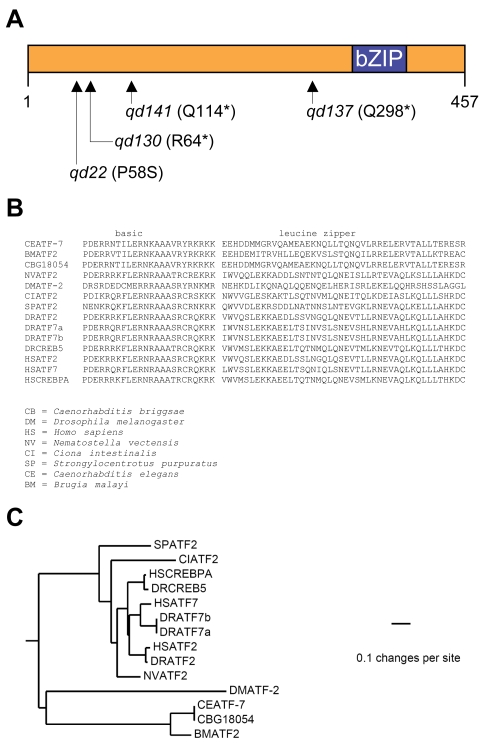
Mutant alleles of *C. elegans atf-7*, an ortholog of the mammalian ATF2/ATF7/CREB5 family of bZIP transcription factors. (A) Identification of mutations in the *C. elegans atf-7* gene. Changes shown in amino acid residues are in reference to the coding region of gene model *C07G2.2a*. (B) Sequence alignment comparing the DNA binding domains of *C. elegans* ATF-7 and human ATF-2. (C) Phylogenetic analysis grouping *C. elegans* ATF-7 with the mammalian ATF2/ATF7/CREB5 family of bZIP transcription factors.

Using single nucleotide polymorphism (SNP)-based mapping [25],[26], we narrowed the region containing the *qd22* mutation to a 250 kb region of the left arm of LG III, where we identified a C→T missense mutation causing a P58S change in the open reading frame defined by the gene *atf-7*, encoding a basic-region leucine zipper (bZIP) domain-containing protein (Figure 2A). Phylogenetic analysis, based on the comparison of the conserved putative DNA binding domain sequence of ATF-7 with other bZIP transcription factors (A.W. Reinke and A.E. Keating, unpublished data), suggests that *C. elegans* ATF-7 is an ortholog of the mammalian ATF2/ATF7/CREB5 family of bZIP transcription factors [27] (Figure 2B and 2C). Injection of the fosmid 25cA04, that includes the *atf-7* locus, resulted in partial rescue of the Esp phenotype (Figure S2).

We were unable to phenocopy the diminished GFP reporter gene expression of the *atf-7(qd22)* mutant by RNAi of the *atf-7* gene in the WT background (Figure S3A), but interestingly, we observed that RNAi of *atf-7* in the *atf-7(qd22)* mutant background resulted in reversion of the diminished GFP reporter gene expression (Figure S3A). RNAi of *atf-7* in WT worms resulted in an Esp phenotype (Figure S3B and Figure S11), but notably, RNAi of *atf-7* in the *atf-7(qd22)* mutant conferred increased pathogen resistance and partial suppression of the Esp phenotype of the *atf-7(qd22)* mutant (Figure S3C and Figure S11). These data suggested that *qd22* is a gain-of-function mutant allele of the *atf-7* gene, which was further corroborated by analysis of the phenotype of the *atf-7(qd22)/atf-7(qd22 qd130)* trans-heterozygote (the *qd130* loss-of-function allele is described below), which is nearly as susceptible to *P. aeruginosa* as the *atf-7(qd22)* mutant (Figure S4 and Figure S12). Interestingly, we observed that the *atf-7(qd22)* allele is recessive with respect to the Esp phenotype (Figure S4).

Based on the evidence that *atf-7(qd22)* was a gain-of-function, and possibly neomorphic, mutant allele of *atf-7*, we anticipated that we would be able to isolate *atf-7* loss-of-function alleles from a screen for suppressors of the attenuated GFP expression phenotype of the *atf-7(qd22)* mutant. We screened 20 000 haploid genomes for mutants with increased GFP reporter expression and isolated an intragenic suppressor of *atf-7(qd22)*, *atf-7(qd22 qd130)*, which carries a nonsense mutation resulting in an early stop codon in the *atf-7* gene (Figure 2A). The *atf-7(qd22 qd130)* mutant allele suppressed the diminished GFP fluorescence phenotype of the *atf-7(qd22)* mutant (Figure 1A), but only partially suppressed the Esp phenotype, demonstrating that the *atf-7* loss-of-function mutant also has an Esp phenotype compared to WT (Figure 1B and Figure S10).

To confirm that the observed Esp phenotype of the *atf-7(qd22 qd130)* mutant was caused by the nonsense mutation in *atf-7*, we also analyzed a second putative null allele of *atf-7*, *atf-7(qd137)* (Figure 2A), which we isolated from a separate screen (described below). The *atf-7(qd137)* mutant exhibited the same Esp phenotype as that observed for the *atf-7(qd22 qd130)* mutant, as well as the trans-heterozygote, *atf-7(qd22 qd130)/atf-7(qd137)* (Figure 1D and Figure S13). We also carried out rescue experiments using a transgene comprised of the genomic *atf-7* locus with GFP fused to the 3′ end between the *atf-7* stop codon and the 3′-untranslated region (UTR). We observed that this transgene partially rescued the Esp phenotype of the *atf-7(qd22 qd130)* mutant (Figure S5). The partial degree of rescue observed for both the *atf-7(qd22)* and *atf-7(qd22 qd130)* mutants may reflect the detrimental effects of overexpression of *atf-7*.

The Esp phenotype of the *atf-7(qd22 qd130)* mutant was consistent with the results of the RNAi experiments (Figure S2B) and suggests that whereas the *atf-7(qd22)* gain-of-function allele confers a strong Esp phenotype, loss of *atf-7* activity also compromises pathogen resistance relative to WT. We observed that the longevity of *atf-7(qd22 qd130)* and *atf-7(qd137)* mutants on *E. coli* OP50 was comparable to that observed for WT (Figure S6 and Figure S16).

### *atf-7* loss-of-function alleles suppress the immunodeficiency phenotype of *pmk-1*

**Figure 3:**
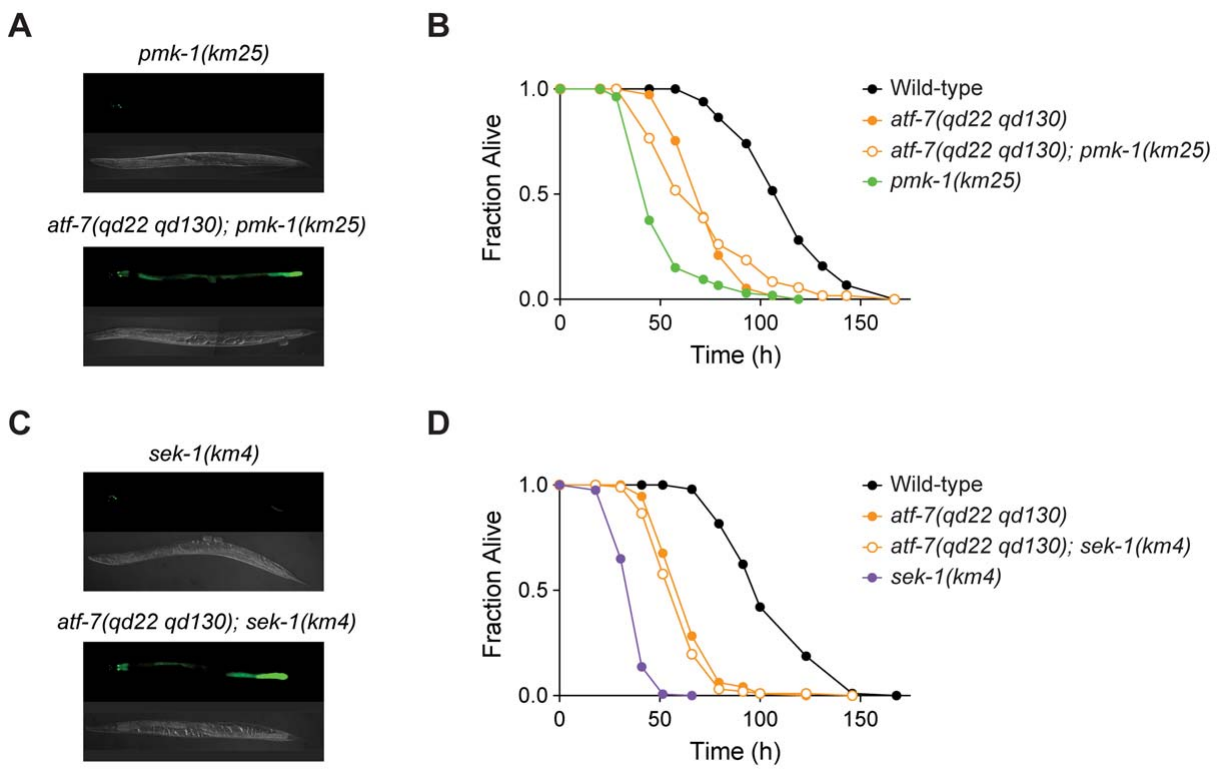
The loss-of-function *atf-7(qd22 qd130)* mutation suppresses the immunodeficient phenotype caused by deficient signaling in the PMK-1 pathway. (A) Fluorescence microscopy images of GFP expression from the *agIs219* transgene in *pmk-1(km25)* and *atf-7(qd22 qd130); pmk-1(km25)* one-day-old adults. (B) Pathogenesis assay of L4 larval stage wild-type worms; *atf-7(qd22 qd130)* and *pmk-1(km25)* mutant animals; and *atf-7(qd22 qd130); pmk-1(km25)* double mutant animals, on *P. aeruginosa* PA14. All strains carry the *agIs219* transgene. The difference in susceptibility between *pmk-1(km25)* mutant animals and *atf-7(qd22 qd130); pmk-1(km25)* double mutant animals is significant (*p*<0.0001). Replicate data can be seen in Figure S14. (C) Fluorescence microscopy images of GFP expression from the *agIs219* transgene in *sek-1(km4)* and *atf-7(qd22 qd130); sek-1(km4)* one-day-old adults. (D) Pathogenesis assay of L4 larval stage wild-type worms; *atf-7(qd22 qd130)* and *sek-1(km4)* mutant animals; and *atf-7(qd22 qd130); sek-1(km4)* double mutant animals, on *P. aeruginosa* PA14. All strains except for KU25 [*sek-1(km4)*] carry the *agIs219* transgene. The difference in susceptibility between *sek-1(km4)* mutant animals and *atf-7(qd22 qd130); sek-1(km4)* double mutant animals is significant (*p*<0.0001). Replicate data can be seen in Figure S15.

Because *atf-7(qd22)* appeared to be a gain-of-function allele of *atf-7* that exhibited the same phenotypes as observed for mutants carrying loss-of-function mutations in PMK-1 pathway components, we hypothesized that ATF-7 might be negatively regulated by the PMK-1 pathway and function as a repressor of the innate immune response. To test this hypothesis, we carried out epistasis analysis using the *atf-7(qd22 qd130)* and *pmk-1(km25)* null alleles. We observed that the *atf-7(qd22 qd130)* loss-of-function allele suppressed the diminished *agIs219* GFP reporter gene expression phenotype of the *pmk-1(km25)* mutant (Figure 3A). Also, the *atf-7(qd22 qd130); pmk-1(km25)* double mutant had a reduced pathogen susceptibility compared to *pmk-1(km25)*, comparable to that of the *atf-7(qd22 qd130)* single mutant (Figure 3B and Figure S14). The partial suppression of the Esp phenotype of the *pmk-1(km25)* mutant by *atf-7(qd22 qd130)* was rescued by a transgene carrying wild-type *atf-7* fused with GFP (Figure S7). Furthermore, we found that *atf-7(qd22 qd130)* also suppressed the pathogen susceptibility (Figure 3D and Figure S15) and diminished *agIs219* GFP reporter expression (Figure 3C) phenotypes of the *sek-1(km4)* mutant.

The Esp phenotypes of the *atf-7(qd22 qd130); pmk-1(km25)* and *atf-7(qd22 qd130); sek-1(km4)* double mutants are comparable to the Esp phenotype of *atf-7(qd22 qd130)* single mutant. The lack of effect of the *pmk-1(km25)* or *sek-1(km4)* mutations on the Esp phenotype in the *atf-7(qd22 qd130)* mutant background is particularly noteworthy in view of the strong Esp phenotype conferred by inactivation of the PMK-1 pathway in the WT background.

Based on the genetic interaction between *atf-7(qd22 qd130)* and *pmk-1(km25)*, we anticipated that a screen aimed at isolating suppressors of the diminished *agIs219* GFP reporter expression and pathogen susceptibility phenotypes of the *pmk-1(km25)* mutant would yield additional loss-of-function alleles of *atf-7*. Indeed, we isolated two more putative null alleles of *atf-7*, the aforementioned *atf-7(qd137)* allele and *atf-7(qd141)* (Figure 2A), from a genetic screen of 15 000 haploid genomes. The suppression of the pathogen susceptibility phenotypes of PMK-1 pathway loss-of-function mutants by *atf-7* null alleles is consistent with a role for ATF-7 downstream of activated PMK-1 in the negative regulation of innate immunity in *C. elegans*.

### Regulation of PMK-1–regulated genes by ATF-7

**Figure 4:**
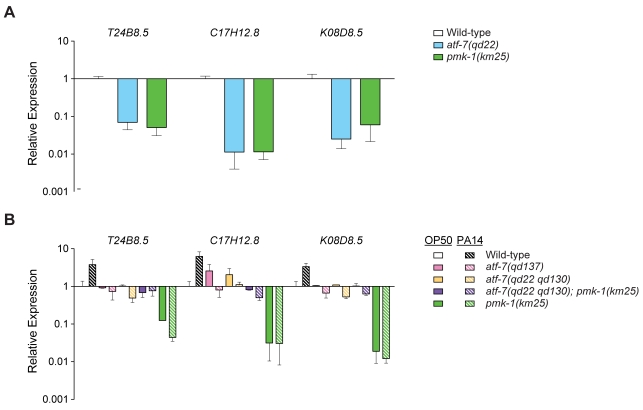
ATF-7 regulation of PMK-1–regulated genes. qRT–PCR analysis of the expression of PMK-1-regulated genes. (A) L4 larval stage worms of the indicated genotype were propagated on *E. coli* OP50 and RNA was prepared as described in the Methods. Expression is relative to wild-type. Shown is the mean from two independent biological replicates with error bars representing SEM. (B) As in (A), except that worms of the indicated geneotype were exposed to *P. aeruginosa* PA14 for 4 h. Expression is relative to wild-type on *E. coli* OP50.

We used quantitative real-time PCR (qRT-PCR) to measure changes in expression of three representative genes previously identified as being regulated by the PMK-1 pathway [22]. We observed that the *atf-7(qd22)* mutant, consistent with the observed effects on *agIs219* GFP reporter expression (Figure 1A), exhibited sharply diminished expression of PMK-1-regulated genes relative to WT on *E. coli* OP50, comparable to the levels observed in the *pmk-1(km25)* mutant (Figure 4A). These data confirm that the observed effects of the *atf-7(qd22)* mutation on *agIs219* expression reflect a change in the regulation of PMK-1-regulated genes.

The basal level of expression of PMK-1-regulated genes, as defined by the levels of expression of genes on the relatively non-pathogenic *E. coli* OP50, was comparable in the *atf-7* loss-of-function mutants and WT (Figure 4B). Confirming the observations with the *agIs219* transgenic reporter, the *atf-7(qd22 qd130)* loss-of-function allele suppressed the markedly diminished basal expression of PMK-1-regulated genes in the *pmk-1(km25)* mutant (Figure 4B). These data are suggestive of a role for ATF-7 in the transcriptional repression of the basal expression of PMK-1-regulated genes, with de-repression of these genes through inhibition of ATF-7 by activated PMK-1. But if ATF-7 functioned solely as a transcriptional repressor of PMK-1-regulated genes, then an increase in basal expression of these genes might be anticipated. However, the basal expression of PMK-1-regulated genes is comparable to the levels observed in WT. This observation, as well as the Esp phenotype of the *atf-7* loss-of-function mutants, is suggestive that ATF-7 functions not only as a repressor of the PMK-1-regulated immune response, but as a positive regulator of innate immunity as well, and thus we sought to examine the requirement for ATF-7 in pathogen-induced gene expression.

Upon exposure to pathogen infection, a number of genes are up-regulated in a PMK-1-dependent manner [22]. We observe that genes that require PMK-1 for induction by *P. aeruginosa* PA14 also require ATF-7 for pathogen-induced expression (Figure 4B). Although the basal expression of PMK-1-regulated genes on *E. coli* OP50 in the *atf-7(qd22 qd130)* and *atf-7(qd137)* mutants is comparable to WT, no induction of expression is observed in the presence of *P. aeruginosa* (Figure 4B). These data also suggest dual switching roles for ATF-7, both as a PMK-1-regulated repressor of the basal expression of PMK-1-regulated genes as well as a PMK-1-dependent activator of PMK-1-regulated genes upon pathogenic *P. aeruginosa* infection. This requirement may contribute to the observed Esp phenotype of the *atf-7(qd22 qd130)* mutant, as optimal regulation of the *C. elegans* innate immune response may be dependent on PMK-1 regulation of ATF-7.

### Phosphorylation of ATF-7 by PMK-1

**Figure 5:**
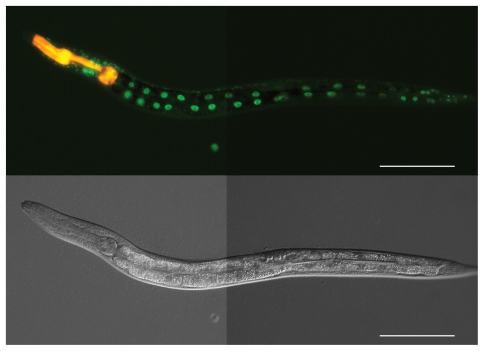
ATF-7 is expressed in the nuclei of intestinal cells in *C. elegans*. Fluorescence and DIC microscopy of a representative L4-staged wild-type worm carrying an *atf-7::GFP* transgene under the regulation of the endogenous genomic *atf-7* promoter and 3′-untranslated region. The red fluorescence from the pharynx is due to a *Pmyo-2::RFP* co-transformation marker. The rescuing capability of this translational fusion transgene was confirmed in both the *atf-7(qd22 qd130)* mutant (Figure S5) and *atf-7(qd22 qd130); pmk-1(km25)* double mutant (Figure S7). Scale bar, 100 µm.

The genetic and gene expression data above are consistent with a role for PMK-1 in the modulation of the transcriptional regulator ATF-7. Because the PMK-1 pathway acts in the intestine in a cell autonomous manner to regulate the innate immune response [21], we anticipated that ATF-7 would also be expressed in the intestine. We observed that a rescuing translational fusion of ATF-7::GFP under the control of the endogenous promoter and 3′ UTR was strongly expressed in the nuclei of intestinal cells (Figure 5).

**Figure 6:**
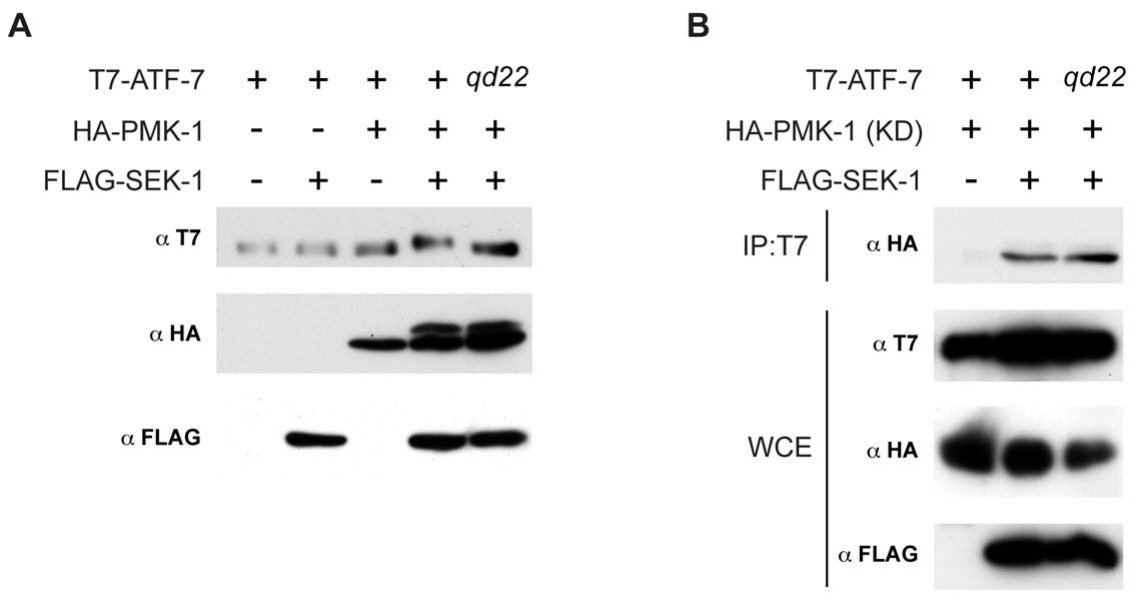
Phosphorylation of ATF-7 by PMK-1. (A) Cos7 cells were transfected with T7-ATF-7 or T7-ATF-7 carrying the P58S *qd22* mutation, along with HA-PMK-1 and FLAG-SEK-1 as indicated. Whole cell extracts were immunoblotted with antibodies that recognize T7 (top), HA (middle), and FLAG (bottom). (B) Cos7 cells were transfected with T7-ATF-7 or T7-ATF-7 carrying the P58S *qd22* mutation, along with kinase-dead (KD) HA-PMK-1 and FLAG-SEK-1 as indicated. ATF-7 was immunoprecipitated with anti-T7 and immunoblotted with anti-HA (top). Whole cell extract were immunoblotted with antibodies that recognize T7 (middle top), HA (middle bottom), and FLAG (bottom).

We next sought to obtain further evidence for a direct interaction between activation of the PMK-1 pathway and ATF-7. We examined whether PMK-1 could phosphorylate ATF-7 by generating activated PMK-1 by co-expressing epitope-tagged *C. elegans atf-7*, *pmk-1*, and *sek-1* cDNAs in Cos7 cells and immunoblotting against T7-ATF-7 to detect changes in gel mobility indicative of phosphorylation. Expression of PMK-1 with SEK-1, which results in activated PMK-1, produced a shift in the T7-ATF-7 protein band indicative of a change in the phosphorylation state (Figure 6A, lane 4). This shift in the ATF-7 band is not seen when either *pmk-1* or *sek-1* cDNAs are not expressed (Figure 6A, lanes 2 and 3), or when ATF-7 is immunoprecipitated and treated with phosphatase (Figure S8). These data are consistent with PMK-1-dependent phosphorylation of ATF-7.

We used a mutated version of PMK-1 that does not have kinase activity to establish that ATF-7 and PMK-1 physically interact. Immunoprecipitation using the T7 antibody, followed by immunoblotting using anti-HA, revealed an HA-PMK-1(kinase-dead)-T7-ATF-7 interaction that was dependent on the activated form of PMK-1, as determined by the requirement for co-transfection of *sek-1* cDNA (Figure 6B).

We introduced the *qd22* mutation into the T7-ATF-7 expressed in Cos7 cells and found that in contrast to the WT ATF-7, the mutant ATF-7 showed no change in gel mobility in the presence of activated PMK-1 (Figure 6A, lane 5), suggestive that the *atf-7(qd22)* allele may encode a form of the protein that can bind PMK-1 (Figure 6B, lane 3), but cannot be phosphorylated by PMK-1. The unusual nature of the *atf-7(qd22)* allele, with respect to the Esp phenotype and effects on PMK-1-regulated gene expression, coupled with the apparent insensitivity of the corresponding mutant ATF-7 protein to PMK-1 activity, further suggests that the phosphorylation of ATF-7 by PMK-1 may function to relieve the transcriptional repressor activity of ATF-7.

### ATF-7 confers immune response specificity to PMK-1 pathway activation

**Figure 7:**
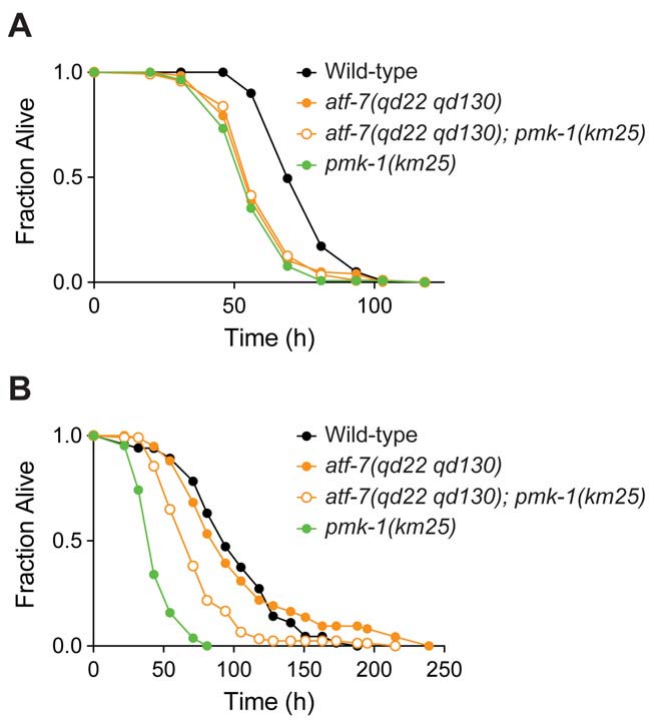
Requirement for ATF-7 in resistance to other bacterial pathogens. (A) Pathogenesis assay of L4 larval stage wild-type worms; *atf-7(qd22 qd130)* and *pmk-1(km25)* mutant animals; and *atf-7(qd22 qd130); pmk-1(km25)* double mutant animals, on *S. marcescens* Db10. All strains carry the *agIs219* transgene. The difference in susceptibility between *atf-7(qd22 qd130)* mutant animals and wild-type worms is significant (*p*<0.0001). (B) Pathogenesis assay of L4 larval stage wild-type worms; *atf-7(qd22 qd130)* and *pmk-1(km25)* mutant animals; and *atf-7(qd22 qd130); pmk-1(km25)* double mutant animals, on *E. faecalis* MMH594. All strains carry the *agIs219* transgene. The difference in susceptibility between *pmk-1(km25)* mutant animals and *atf-7(qd22 qd130); pmk-1(km25)* double mutant animals is significant (*p*<0.0001). There is no difference in susceptibility between *atf-7(qd22 qd130)* mutant animals and wild-type worms (*p*>0.9).

In order to determine whether our observations were specific to infection by *P. aeruginosa* PA14, we examined the role of ATF-7 in pathogen resistance to two other microbial pathogens that cause lethal infections in *C. elegans*, *Serratia marcescens* and *Enterococcus faecalis*. On *S. marcescens* Db10, the *pmk-1(km25)* mutant had a weak Esp phenotype (Figure 7A) compared to the strong Esp phenotype exhibited on *P. aeruginosa* PA14 (Figure 3B). The *atf-7(qd22 qd130)* mutant also had a similarly weak phenotype, as did the *atf-7(qd22 qd130); pmk-1(km25)* double mutant (Figure 7A). These data suggest that the PMK-1 pathway and ATF-7 are required for resistance to *S. marcescens*, but the comparable Esp phenotypes of single mutants and the *atf-7(qd22 qd130); pmk-1(km25)* double mutant are consistent with PMK-1 and ATF-7 functioning as positive regulators of pathogen resistance in the same pathway, or with ATF-7 under negative regulation by PMK-1 with ATF-7 functioning as a transcriptional repressor.

Interestingly, on the Gram-positive pathogen *E. faecalis* MMH594, the *atf-7(qd22 qd130)* mutant does not have an appreciable Esp phenotype (Figure 7B), suggestive that ATF-7 may not serve as a positive regulator of resistance to *E. faecalis*. In addition, the *atf-7(qd22 qd130)* mutation only partially suppresses the Esp phenotype of the *pmk-1(km25)* mutant (Figure 7B), suggestive that there are both ATF-7-dependent and ATF-7-independent mechanisms downstream of PMK-1 in response to *E. faecalis*. Distinct sets of genes have been observed to be induced by exposure to different bacteria, and little is known about how the transcriptional responses to Gram-positive bacteria and Gram-negative bacteria differ [28]. Of note, we observed diminished GFP expression from the *agIs219* transgene on Gram-positive bacteria relative to expression on *E. coli* OP50 (D.H.K., unpublished data). Gene expression studies of genes induced in *C. elegans* infection with Gram-positive bacteria, and the identification of such genes that are regulated by the PMK-1 pathway may further illuminate the differences in the role of ATF-7 observed between *E. faecalis* and *P. aeruginosa*.

**Figure 8:**
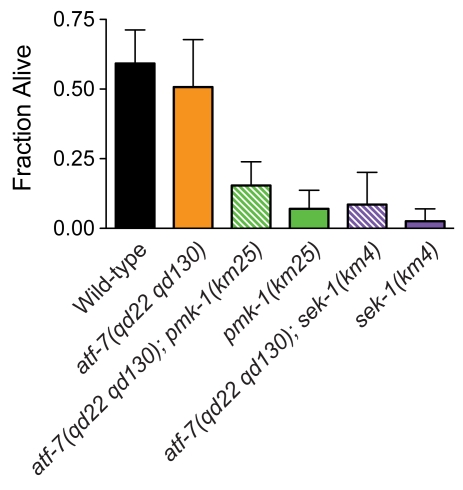
ATF-7 does not contribute to arsenite resistance in *C. elegans*. Arsenite stress assay of L4 larval stage wild-type worms; *atf-7(qd22 qd130), pmk-1(km25)*, and *sek-1(km4)* mutant animals; and *atf-7(qd22 qd130); pmk-1(km25)* and *atf-7(qd22 qd130); sek-1(km4)* double mutant animals. Shown is the fraction of worms alive after 18 h. Error bars are standard deviation. The differences in survival between *atf-7(qd22 qd130)* mutant animals and *atf-7(qd22 qd130); pmk-1(km25)* double mutant animals, and *atf-7(qd22 qd130)* mutant animals and *atf-7(qd22 qd130); sek-1(km4)* double mutant animals are significant (*p*<0.05 for each comparison).

The PMK-1 pathway regulates the response to arsenite and oxidative stress through regulation of the transcription factor SKN-1 [29],[30]. We observed that the *atf-7(qd22 qd130)* mutant did not exhibit enhanced sensitivity to arsenite stress, and in addition, did not suppress the arsenite sensitivity of the *pmk-1(km25)* mutant or the *sek-1(km4)* mutant (Figure 8). In addition, we observed that *skn-1* mutants did not exhibit enhanced susceptibility to *P. aeruginosa* (Figure S9). Whereas PMK-1 mediates multiple responses to environmental stressors, including oxidative stress and pathogen infection, these data suggest that the transcription factor substrates of PMK-1, ATF-7 and SKN-1, confer specificity to PMK-1-mediated responses.

## Discussion

We have described the identification and characterization of ATF-7, a *C. elegans* ortholog of the mammalian ATF2/ATF7/CREB5 family of bZIP transcription factors, as a transcriptional regulator of PMK-1-mediated innate immunity in *C. elegans*. We isolated four mutant alleles of *C. elegans atf-7* from three different forward genetic screens. First, the gain-of-function *qd22* allele was isolated from a large-scale screen for mutants with diminished PMK-1-dependent GFP reporter gene expression and an Esp phenotype. The isolation of *qd22* served as a starting point for the characterization of ATF-7, and the analysis of this unusual gain-of-function allele provided insights into the mechanism of ATF-7 regulation by PMK-1. The increased levels of PMK-1 activation in the *atf-7(qd22)* mutant relative to WT (Figure 1C) may reflect feedback loops that serve to counteract the suppression of the PMK-1-mediated transcriptional response by increasing levels of activated PMK-1. Although *atf-7(qd22)* acts as a gain-of-function allele, we determined that *atf-7(qd22)* is recessive with regard to pathogen susceptibility (Figure S4). We suggest that whereas the *qd22* mutant ATF-7 protein cannot be phosphorylated by PMK-1 and thus functions as a constitutive repressor (Figure 6A), the resulting protein may undergo changes in structure and folding that compromise the ability of the mutant ATF-7 to compete with WT ATF-7 at corresponding promoter sites in the *atf-7(qd22)/atf-7(+)* trans-heterozygote.

Based on evidence that *atf-7(qd22)* was a gain-of-function allele, we isolated the *atf-7(qd22 qd130)* allele as an intragenic suppressor of *atf-7(qd22)*. A third genetic screen aimed at isolating suppressors of *pmk-1(km25)* yielded two additional putative null alleles of *atf-7*, *qd137* and *qd141*. The genetic analysis of loss-of-function alleles of *atf-7* allowed us to begin to address the physiological role of ATF-7 in innate immunity. Genetic interaction analysis of *atf-7* mutant alleles suggests that PMK-1 negatively regulates ATF-7, which in turn functions as a negative regulator of *C. elegans* innate immunity to *P. aeruginosa* and *E. faecalis*. At the same time, the Esp phenotype of *atf-7* loss-of-function mutants and the analysis of *P. aeruginosa*-induced gene expression were suggestive of a requirement for ATF-7 in the activation of the inducible innate immune response, as ATF-7 was shown to be required for the increased expression of PMK-1-regulated genes in response to *P. aeruginosa* infection. Interestingly, the lack of an Esp phenotype of *atf-7(qd22 qd130)* on *E. faecalis* may be suggestive of the absence of a PMK-1-regulated inducible response to *E. faecalis* that is regulated by ATF-7.

**Figure 9:**
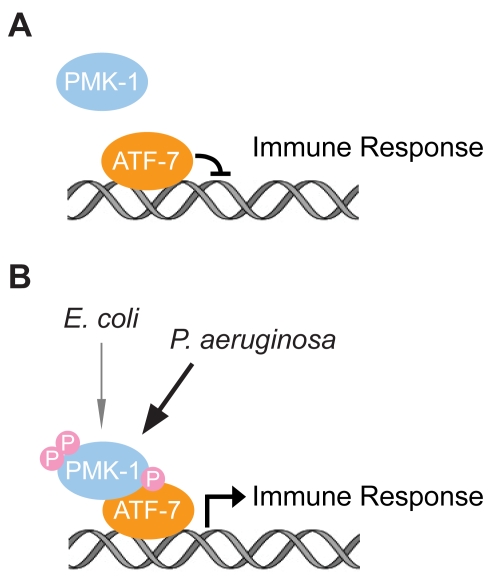
Model for the function of ATF-7 in *C. elegans* innate immunity. (A) Repression of PMK-1–regulated immune effector gene expression by ATF-7 in the absence of PMK-1 activation. (B) Activation of basal and pathogen-induced immune effector gene expression by PMK-1 phosphorylation of ATF-7, which switches ATF-7 from a repressor to an activator of transcription.

We showed that ATF-7 physically interacts with activated PMK-1 and undergoes PMK-1-dependent phosphorylation in mammalian cells in heterologous expression assays. Based on these data, we propose that activation of the PMK-1 pathway in response to pathogen infection results in PMK-1 phosphorylation of ATF-7, leading to a switch in the activity of ATF-7 from transcriptional repressor to an activator that facilitates *P. aeruginosa*-induced gene expression (Figure 9). In yeast, phosphorylation of the CREB/ATF transcription factor Sko1p downstream of the ancestral Hog1p MAPK pathway in response to osmotic stress converts Sko1p from a transcriptional repressor to an activator [31]. Our data suggest that this mode of transcriptional regulation by MAPK activation has been conserved in *C. elegans* innate immunity. Further work may define the detailed mechanisms by which ATF-7 transcriptional control is modulated by PMK-1.

In view of the multiple substrates for p38 MAPK that have been established in mammalian cell systems and the multiple activities of p38 MAPK in mammalian innate immunity, it is perhaps surprising that loss of activity of a single transcription factor, the *C. elegans* ortholog of mammalian ATF2, is sufficient to suppress the immunocompromised phenotype caused by loss of p38 MAPK activity in *C. elegans*. Mice homozygous for a deletion of the *ATF2* gene die shortly after birth due to the lack of pulmonary surfactant [8], although analysis of cells from mutant mice expressing low levels of ATF2 are suggestive of a role for ATF2 in the modulation of cytokine expression [32]. Interestingly, these studies also indicate a role for mammalian ATF2 in both activating and inhibitory activities on the immune response [32]. Whether ATF2 plays a correspondingly homologous role in mammalian innate immunity downstream of p38 MAPK, paralleling the activity of *C. elegans* ATF-7, awaits the further molecular genetic analysis of the ATF2/ATF7/CREB5 family in mammalian systems. Our data establish the SARM-p38 MAPK-ATF-7 pathway as a major pathway of *C. elegans* innate immunity and suggest that the regulation of ATF2/ATF7 family of bZIP transcription factors by p38 MAPK may represent a key feature of innate immunity in ancestral organisms that was retained even as Toll-dependent NF-kB immune signaling was lost [33]. The mechanism of regulation of ATF-7 activity by PMK-1 may also provide insights into conserved mechanisms by which p38 MAPK modulates the activity of ATF2/ATF7 in mammalian innate immunity.

## Materials and Methods

### *C. elegans* strains

*C. elegans* was maintained and propagated on *E. coli* OP50 as described [34]. AU78, an N2-derived strain carrying the *agIs219* transgene was used as the wild-type strain [21]. CB4856 was used for single nucleotide polymorphism-based mapping [26].

Previously isolated and characterized mutants used: LG II: *nsy-1(ag3)* [10], *nsy-1(ky397)* [35]. LG III: *tir-1(qd4)* [21]. LG IV: *pmk-1(km25)* [36], *skn-1(zu67)*, *skn-1(zu135)* [37]. LG X: *sek-1(km4)* [10],[38].

Mutants described in this study: ZD442 [*agIs219 atf-7(qd22) III*] was isolated as described below and backcrossed three times to its parental strain, AU78. ZD318 [*agIs219 atf-7(qd22 qd130) III*] was isolated as described below and outcrossed four times to wild-type strain N2. ZD39 [*agIs219 III; pmk-1(km25) IV*] was made by crossing the *agIs219* transgene from strain AU78 into *pmk-1(km25)*. ZD395 [*agIs219 III; sek-1(km4) X*] was made by crossing the *agIs219* transgene from strain AU78 into *sek-1(km4)*. ZD332 [*agIs219 atf-7(qd137) III; pmk-1(km25) IV*] and ZD402 [*agIs219 atf-7(qd141) III; pmk-1(km25) IV*] were isolated as described below. ZD332 was backcrossed three times to ZD39. ZD350 [*agIs219 atf-7(qd137) III*] was made by removing *pmk-1(km25)* from *agIs219 atf-7(qd137); pmk-1(km25)*, which had been previously backcrossed twice to ZD39, by outcrossing to N2. ZD326 [*agIs219 atf-7(qd22 qd130) III; pmk-1(km25) IV*] was made by crossing *pmk-1(km25)* into *agIs219 atf-7(qd22 qd130)* and was outcrossed to *pmk-1(km25)* an additional three times. ZD340 [*agIs219 atf-7(qd22 qd130) III; sek-1(km4) X*] was made by crossing *sek-1(km4)* into *agIs219 atf-7(qd22 qd130)* and was outcrossed to *sek-1(km4)* an additional three times.

### Pathogenesis assays

Pathogenesis assays with *P. aeruginosa* PA14 [39], *S. marcescens* Db10 [40] and *E. faecalis* MMH594 [41],[42] were performed as described previously with the following modifications. Single colonies of *P. aeruginosa* PA14 and *S. marcescens* Db10 were used to inoculate 3 ml cultures of Luria-Bertani (LB) broth, which were then incubated overnight at 37°C. Five microliters of the *S. marcescens* Db10 culture was used to seed standard 35-mm slow-kill assay plates, whereas five microliters of the *P. aeruginosa* PA14 culture was used to seed 35-mm slow-kill assay plates containing 0.05 mg/ml 5-fluorodeoxyuridine (FUDR), used to prevent eggs from hatching. Seeded plates were incubated at 37°C overnight and then incubated at room temperature overnight. A single colony of *E. faecalis* MMH594 was used to inoculate a 3 ml culture of brain heart infusion (BHI) broth containing 80 µg/ml of kanamycin, which was then incubated at 37°C for 5 hours. Seven microliters of culture was used to seed 35-mm BHI agar plates containing 80 µg/ml of kanamycin, which were incubated at 25°C overnight. In all pathogenesis assays, the size of the bacterial lawn was small, meaning that the culture was seeded in the middle of the plate and was not spread to the edge. For each assay, approximately 20–40 L4-staged worms were picked over to prepared plates, with 3–5 plates per strain. The sample sizes for each assay are provided in Table S1. All pathogenesis assays were conducted at 25°C. Plates were checked at regular intervals for survival and worms that did not respond to gentle prod from a platinum wire were scored as dead. Worms on *S. marcescens* plates were transferred to new plates on days 1, 2, and 3 of the assay. All *S. marcescens* plates in a single assay were seeded on the same day. Worms on *P. aeruginosa* PA14 plates containing FUDR and *E. faecalis* MMH594 plates did not require transferring. Statistical analyses of survival curves were performed in Prism 5 (GraphPad) using the log-rank test function, which computes the Mantel-Haenszel method.

### Isolation of mutants with diminished *agIs219* reporter expression and enhanced susceptibility to killing by *P. aeruginosa* PA14

Mutagenesis using ethyl methane sulfonate (EMS) was carried out following standard methods [43]. The synchronized F_2_ generation L1 stage larvae were plated onto NGM plates seeded with *E. coli* OP50 and incubated for 55 hours at 20°C and subsequently sorted with a Union Biometrica COPAS Biosorter. Worms with diminished fluorescence compared to wild-type worms were directly plated onto a plate seeded with *P. aeruginosa* PA14, and incubated at 25°C. The plates were screened at 24 hours for dead worms. Following the rationale of our previously reported screen for Esp mutants [10], carcasses of dead worms were picked to individual NGM plates seeded with *E. coli* OP50, allowing the fertilized eggs inside each carcass to hatch so that the mutant strains could be recovered. In three separate screens a total of 140 000 haploid genomes were mutagenized. We note that the yield of the screen is strongly dependent on the time at which the Esp screening takes place, and that the 24 h time point represented a particularly stringent time such that siblings were rarely isolated among the mutant isolates. The early time of screening also accounts for the relatively low yield of mutants from the number of genomes mutagenized and the high specificity of isolated mutations for the PMK-1 pathway.

Single-nucleotide polymorphism (SNP)-based mapping using the *C. elegans* isolate CB4856 was performed as reported [26] with modifications utilizing SNPs that were analyzed by the DraI restriction enzyme for the rapid rough mapping of mutant isolates [25]. Once chromosomal linkage was determined, complementation testing was performed using previously isolated mutant alleles (*tir-1(qd4)*, *nsy-1(ag3)* and *nsy-1(ky397)*, *sek-1(km4)*, and *pmk-1(km25)*). After assignment of the isolated alleles into complementation groups, the open reading frame of the affected gene was sequenced to identify the causative mutation in each allele. Isolates from the screen and the identified mutations are shown in Table 1. Using this approach, a single mutant allele not corresponding to previously identified mutants, *qd22*, was isolated. Fine mapping of *qd22* was carried out using CB4856 SNP-based mapping. The location of *qd22* on the left arm of LG III was in the vicinity of the *agIs219* integrated transgenic array, and thus a strain carrying *qd22* without *agIs219* was generated and SNP mapping was carried out using the Esp phenotype of *qd22*. In order to facilitate interpretation of the pathogen killing assays with recombinants, a strain carrying the *qd22* mutation (without the *agIs219* transgene) and the CB4856-derived allele of *npr-1*, 215F, was utilized for crossing with CB4856 because of the enhanced susceptibility conferred by the 215F allele of *npr-1* relative to the Bristol N2 background [44],[45] in which *qd22* was initially isolated.

### Isolation of *atf-7(qd22 qd130)*

A forward genetic screen to identify suppressors of the *atf-7(qd22)* diminished *agIs219* GFP fluorescence phenotype was carried out similarly as above. Briefly, *C. elegans atf-7(qd22)* hermaphrodites carrying *agIs219* were mutagenized with EMS and synchronized larvae of the F_2_ generation were plated onto NGM plates seeded with *E. coli* OP50 and incubated for 55 hours at 20°C. The F_2_ worms were screened for GFP expression from the *agIs219* transgene using a Zeiss Stereo V12 Discovery microscope with a GFP wide-band fluorescence cube. Any F_2_ worm with increased fluorescence compared to *atf-7*(*qd22*) was singled to a NGM plate seeded with *E. coli* OP50. Isolates with increased fluorescence were then tested for suppression of the *atf-7(qd22)* Esp phenotype to *P. aeruginosa* PA14 using the PA14 pathogenesis assay described above. The *atf-7* coding region was then sequenced in isolates that had both increased fluorescence and diminished pathogen susceptibility.

### Isolation of *atf-7(qd137)* and *atf-7(qd141)*

A genetic screen for suppressors of the *pmk-1(km25)* mutant was carried out using ZD39 as the starting strain and following a procedure as outlined for the isolation of *atf-7(qd22)* suppressor mutants.

### Generation of transgenic animals

*C. elegans* genomic fosmids (Geneservice) were isolated using Qiagen Miniprep Kits following the standard protocol. Fosmid 24cA04 was injected into ZD442 at a concentration of 20 ng/µl, along with 25 ng/µl of *Pmyo-2::RFP* as a co-injection marker and 55 ng/µl of pBlueScript (Stratagene) as carrier DNA. The fluorescently-tagged *atf-7* construct was generated by yeast-mediated ligation of genomic fragments generated by PCR using fosmid 25cA04 as template DNA and Phusion high-fidelity DNA polymerase (New England Biolabs). A 22794 bp genomic region, from 7474 to 30267 with respect to fosmid 25cA04, was covered in the fluorescently-tagged *atf-7* construct. This construct was injected into ZD326 at a concentration of 20 ng/µl, along with 32 ng/µl of *Pmyo-2::RFP* as a co-injection marker and 10 ng/µl of pBlueScript (Stratagene) as carrier DNA. Two independent lines carrying this construct were crossed into strain ZD318 using standard genetic techniques.

### Yeast-mediated ligation construction of *atf-7::GFP*

Yeast-mediated ligation of *atf-7::GFP* was performed as previously described [46]. Briefly, the 22794 bp operonic region containing the *atf-7* gene was amplified in fragments ranging in size from ∼1 kb to 4 kb in 8 separate PCR reactions with at least a 50 bp overlap between adjacent fragments. The gene encoding GFP was amplified from the Fire vector pPD95.75 [47]. The 9 PCR products, along with destination vector pRS426 (ATCC) digested with BamH1 and Xho1 restriction enzymes (New England Biolabs), were transformed into yeast strain FY2 following standard procedures. Phenol-chloroform extraction was used to isolate yeast DNA, which was then transformed into DH5-α electrocompetent cells (Invitrogen) and isolated using Qiagen Miniprep Kits following the standard protocol.

### Quantitative real-time PCR

Synchronized populations of wild-type and indicated mutant strains were grown to the L4 larval stage. For *P. aeruginosa* exposure experiments, L4 stage worms were washed onto plates seeded with *E. coli* OP50 or *P. aeruginosa* PA14 in parallel, dried, and incubated for four hours at 25°C. Samples were collected, frozen in liquid nitrogen, and stored at −80°C before RNA extraction using TRI reagent (Ambion). cDNA was prepared with the Retroscript kit (Ambion) using oligo dT primers. The reverse transcription reaction was also performed without reverse transcriptase for each sample, and subsequent qRT-PCR on these control reactions showed that no contaminating genomic DNA was present. qRT-PCR was performed with a Mastercycler Realplex (Eppendorf) with SYBR Green detection in triplicate reactions of 20 µl. All primers were previously reported, and relative expression between samples was determined using *snb-1* as a reference gene [22]. Fold change was calculated using the Pfaffl method [48]. Standardization between two biological replicates was performed as described [49].

### RNAi of *atf-7*

A 771 bp segment of the *atf-7* coding region, corresponding to bases 11532 to 12303 with respect to cosmid C07G2, was amplified by PCR and subcloned into the Fire vector L4440. RNAi by bacterial feeding using *E. coli* HT115 bacteria expressing either the L4440-derived *atf-7* RNAi vector or the empty L4440 vector (control RNAi) was carried out as reported [50]. L4 animals were fed on RNAi bacteria plates, and the F_1_ generation animals were assayed for susceptibility to *P. aeruginosa* PA14 or analyzed for GFP expression from the *agIs219* transgene.

### Immunoblotting of *C. elegans* lysates

Immunoblotting against *C. elegans* PMK-1 and activated PMK-1 (Promega) was carried out as described previously [10].

### Visualization of *agIs219* GFP reporter gene expression and fluorescently-tagged *atf-7*

To visualize expression of the *agIs219* reporter, L4-staged worms, grown at 20°C, were picked over to normal maintenance plates and placed at 20°C overnight. After approximately 18 h, worms were mounted on 2% agarose pads and immobilized in 10 mM sodium azide. Slides were viewed using an AxioImager Z1 fluorescence microscope (Zeiss) with an A-Plan 10X/0.25 objective (Zeiss) and pictures were taken using an AxioCam HRm camera. To visualize expression of fluorescently-tagged *atf-7*, L4-staged worms, grown on NGM agar plates seeded with *E. coli* OP50 at 20°C, were mounted and imaged as described above with a Plan-Apochromat 20X/0.8 objective (Zeiss). Background intestinal autofluorescence was removed by taking a picture with the DAPI filter and subtracting the resulting picture from the image taken with the GFP filter.

### Expression in Cos7 cells, immunoprecipitation, and immunoblotting

Cos7 cells were maintained in DMEM supplemented with 10% fetal calf serum, 100 µg/ml penicillin G and 100 µg/ml streptomycin at 37°C and 5% CO_2_. Cos7 cells (1×10^6^) were plated in 6-cm dishes and transfected with a total of 6 µg DNA containing various expression vectors by using FuGENE6 (Roshe). The ATF-7 expression vector contained the *atf-7c* isoform. After 48 h, cells were collected and washed once with ice-cold phosphate-buffered saline (PBS) and lysed in 0.6 ml of extraction buffer (20 mM HEPES, pH 7.4, 150 mM NaCl, 1.5 mM MgCl_2_, 2 mM EGTA, 2 mM dithiothreitol, 1 mM phenylmethylsulfonyl fluoride, 1.7 µg/ml aprotinin and 0.5% Triton X-100). Cellular debris was removed by centrifugation at 10 000×*g* for 5 min. Small aliquots of each cell lysate were boiled with SDS-sample buffer and were used as whole cell extracts. Remaining cell lysates were divided into 200 µl and each cell lysate was incubated with 0.5 µg of various antibodies and 10 ml protein G-Sepharose (Amersham Biosciences, Piscataway, NJ). The immune complexes were washed five times with wash buffer (20 mM HEPES, pH 7.4, 150 mM NaCl) and then boiled with SDS-sample buffer. Phosphatase treatment was performed on immunoprecipitated samples with Lambda protein phosphatase (New England Biolabs) at 30°C for 10 minutes. Immunoblotting was performed as described previously [51].

### Arsenite stress assays

Sensitivity of mutant strains to oxidative stress was determined using sodium arsenite. Briefly, mutant worms were grown on *E. coli* OP50. L4-staged animals were transferred to standard slow-killing plates supplemented with 5 mM sodium arsenite and 0.05 mg/ml of FUDR, seeded with concentrated *E. coli* OP50. The sample sizes for the arsenite stress assay are provided in Table S1. Stress assays were performed at 20°C. Animals were considered dead when they no longer responded to a gentle prod with a platinum wire. Statistical analysis of data was performed in Prism 5 (GraphPad) using an unpaired, two-tailed, Student's *t*-test.

### Lifespan assays

Strains used in the lifespan assays were maintained on *E. coli* OP50 at 20°C. Approximately 40 L4-staged worms (Day 0) were picked over to NGM plates containing 0.05 mg/ml of FUDR, seeded with *E. coli* OP50. Four to five plates for each strain were used in each experiment and plates that had become contaminated or plates in which the worms had borrowed were excluded upon the appearance of contamination/borrowing. Worms that had protruding/exploding vulvas and worms that crawled off the plate were censored. The sample sizes for each assay are provided in Table S1. Lifespan assays were performed at 20°C. Animals were considered dead when they no longer responded to a gentle prod with a platinum wire.

## Supporting Information

Figure S1 Lifespan of *atf-7(qd22)* and WT worms on *E. coli* OP50. Lifespan assay of L4 larval stage wild-type worms and *atf-7(qd22)* mutant animals on *E. coli* OP50. Both strains carry the *agIs219* transgene. Replicate data can be seen in Figure S16.
(0.13 MB PDF)

Figure S2 Rescue of *atf-7(qd22)* Esp phenotype. Pathogenesis assay of L4 larval stage wild-type worms, *atf-7(qd22)* mutant animals, and three independent transgenic lines (*qdEx14*, *qdEx15*, and *qdEx16*) of *atf-7(qd22)* mutant animals carrying fosmid 25cA04. All strains carry the *agIs219* transgene. The difference in susceptibility between *atf-7(qd22)* mutant animals and each transgenic line carrying fosmid 25cA04 is significant (*p*<0.0001).
(0.13 MB PDF)

Figure S3 *atf-7(qd22)* is a gain-of-function allele. (A) Fluorescence microscopy images of GFP expression from the *agIs219* transgene in wild-type worms and *atf-7(qd22)* mutant worms each exposed to both control RNAi and RNAi of *atf-7*. (B) Pathogenesis assay comparing the effects of control RNAi and RNAi of *atf-7* on survival of wild-type worms on *P. aeruginosa* PA14. The difference in susceptibility between wild-type worms treated with control RNAi and *atf-7* RNAi is significant (*p*<0.0001). Replicate data can be seen in Figure S11. (C) Pathogenesis assay comparing the effects of control RNAi and RNAi of *atf-7* on survival of *atf-7(qd22)* mutant animals on *P. aeruginosa* PA14. The difference in susceptibility between *atf-7(qd22)* mutant animals treated with control RNAi and *atf-7* RNAi is significant (*p*<0.0001). Replicate data can be seen in Figure S11.
(0.30 MB PDF)

Figure S4 *atf-7(qd22)* confers a recessive Esp phenotype. Pathogenesis assay of L4 larval stage wild-type worms; *atf-7(qd22)* and *atf-7(qd22 qd130)* mutant animals; and *atf-7(qd22)/atf-7(+)* and *atf-7(qd22)/atf-7(qd22 qd130)* trans-heterozygotes, on *P. aeruginosa* PA14. All strains carry the *agIs219* transgene. The differences in susceptibility between *atf-7(qd22)* mutant animals and *atf-7(qd22)/atf-7(+)* trans-heterozygotes, and *atf-7(qd22 qd130)* mutant animals and *atf-7(qd22)/atf-7(qd22 qd130)* trans-heterozygotes are significant (*p*<0.0001 for each comparison). Replicate data can be seen in Figure S12.
(0.13 MB PDF)

Figure S5 Expression of *atf-7::GFP* rescues the *atf-7(qd22 qd130)* Esp phenotype. Pathogenesis assay of wild-type worms, *atf-7(qd22 qd130)* mutant animals, and two independent transgenic lines (*qdEx17* and *qdEx19*) of *atf-7(qd22 qd130)* mutant animals carrying the *atf-7::GFP* construct under the control of the endogenous *atf-7* genomic promoter and 3′- untranslated region, on *P. aeruginosa* PA14. All strains carry the *agIs219* transgene. The difference in susceptibility between *atf-7(qd22 qd130)* mutant animals and each transgenic line carrying the *atf-7::GFP* transgene is significant (*p*<0.0001 for each comparison).
(0.13 MB PDF)

Figure S6 Lifespan of *atf-7* loss-of-function mutants and WT worms on *E. coli* OP50. Lifespan assay of L4 larval stage wild-type worms, *atf-7(qd22 qd130)* and *atf-7(qd137)* mutant animals on *E. coli* OP50. All strains carry the *agIs219* transgene. Replicate data can be seen in Figure S16.
(0.13 MB PDF)

Figure S7 Expression of *atf-7::GFP* rescues the *atf-7(qd22 qd130)* suppression of *pmk-1(km25)*. Pathogenesis assay of *pmk-1(km25)* mutant animals and *atf-7(qd22 qd130); pmk-1(km25)* double mutant animals, along with three independent transgenic lines (*qdEx17*, *qdEx18*, and *qdEx19*) of *atf-7(qd22 qd130); pmk-1(km25)* double mutant animals carrying the *atf-7::GFP* construct under the control of the endogenous *atf-7* genomic promoter and 3′-untranslated region, on *P. aeruginosa* PA14. All strains carry the *agIs219* transgene. The difference in susceptibility between *atf-7(qd22 qd130); pmk-1(km25)* double mutant animals and each transgenic line carrying the *atf-7::GFP* transgene is significant (*p*<0.0001 for each comparison).
(0.13 MB PDF)

Figure S8 PMK-1 phosphorylation of ATF-7 is sensitive to phosphatase. Cos7 cells were transfected with T7-ATF-7, HA-PMK-1, and FLAG-SEK-1 as indicated. ATF-7 was immunoprecipitated with anti-T7, treated with phosphatase where indicated, and immunoblotted with anti-T7 (top). Whole cell extracts were immunoblotted with antibodies that recognize HA (middle) and FLAG (bottom).
(0.20 MB PDF)

Figure S9 *skn-1* mutants do not exhibit an Esp phenotype. Pathogenesis assay of L4 larval stage wild-type worms, *skn-1(zu67)* and *skn-1(zu135)* mutant animals on *P. aeruginosa* PA14.
(0.13 MB PDF)

Figure S10 Replicate of pathogenesis assay shown in Figure 1B. Chart and bar graphs showing the LT_50_ means, LT_50_ standard deviations (S.D.), and sample sizes from two independent *P. aeruginosa* pathogenesis assays with wild-type worms, *atf-7(qd22)* and *atf-7(qd22 qd130)* mutant animals.
(0.18 MB PDF)

Figure S11 Replicate of pathogenesis assay shown in Figure S3B and S3C. Chart and bar graphs showing the LT_50_ means, LT_50_ standard deviations (S.D.), and sample sizes from two independent *P. aeruginosa* pathogenesis assays with wild-type worms treated with control RNAi and *atf-7* RNAi, and *atf-7(qd22)* mutant animals treated with control RNAi and *atf-7* RNAi.
(0.19 MB PDF)

Figure S12 Replicate of pathogenesis assay shown in Figure S4. Chart and bar graphs showing the LT_50_ means, LT_50_ standard deviations (S.D.), and sample sizes from two independent *P. aeruginosa* pathogenesis assays with wild-type worms; *atf-7(qd22)* and *atf-7(qd22 q130)* mutant animals; and *atf-7(qd22)/atf-7(+)*, *atf-7(qd22 qd130)/atf-7(+)*, and *atf-7(qd22)/atf-7(qd22 qd130)* trans-heterozygotes.
(0.20 MB PDF)

Figure S13 Replicate of pathogenesis assay shown in Figure 1D. Chart and bar graphs showing the LT_50_ means, LT_50_ standard deviations (S.D.), and sample sizes from two independent *P. aeruginosa* pathogenesis assays with wild-type worms; *atf-7(qd22 q130)* and *atf-7(qd137)* mutant animals; and *atf-7(qd22 qd130)/atf-7(+)*, *atf-7(qd137)/atf-7(+)*, and *atf-7(qd22 qd130)/atf-7(qd137)* trans-heterozygotes.
(0.20 MB PDF)

Figure S14 Replicate of pathogenesis assay shown in Figure 3B. Chart and bar graphs showing the LT_50_ means, LT_50_ standard deviations (S.D.), and sample sizes from two independent *P. aeruginosa* pathogenesis assays with wild-type worms; *atf-7(qd22 q130)* and *pmk-1(km25)* mutant animals; and *atf-7(qd22 qd130); pmk-1(km25)* double mutant animals.
(0.19 MB PDF)

Figure S15 Replicate of pathogenesis assay shown in Figure 3D. Chart and bar graphs showing the LT_50_ means, LT_50_ standard deviations (S.D.), and sample sizes from two independent *P. aeruginosa* pathogenesis assays with wild-type worms; *atf-7(qd22 q130)* and *sek-1(km4)* mutant animals; and *atf-7(qd22 qd130); sek-1(km4)* double mutant animals.
(0.19 MB PDF)

Figure S16 Replicate of lifespan assay shown in Figure S1 and Figure S6. Chart and bar graphs showing the LT_50_ means, LT_50_ standard deviations (S.D.), and sample sizes from two independent lifespan assays with wild-type worms, *atf-7(qd22)*, *atf-7(qd22 q130)*, and *atf-7(qd137)* mutant animals.
(0.18 MB PDF)

Table S1 Sample sizes for each pathogenesis, arsenite stress, and lifespan assay. Chart showing the sample sizes for each pathogenesis, arsenite stress, and lifespan assay presented in this work. Sample size does not include censored worms.
(0.16 MB PDF)
