# Supplementary figures and images for: Phosphorylation of the Conserved Transcription Factor ATF-7 by PMK-1 p38 MAPK Regulates Innate Immunity in Caenorhabditis elegans
Source: PLoS Genet. 2010 Apr 1;6(4):e1000892. doi: 10.1371/journal.pgen.1000892 (PMC2848548; doi:10.1371/journal.pgen.1000892)

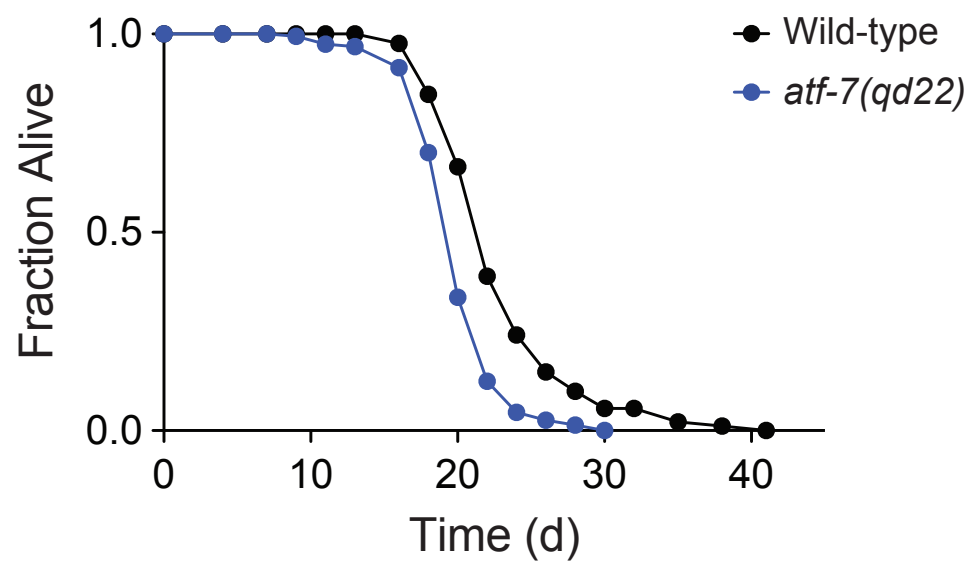

Supplement: Figure S1 — Lifespan of atf-7(qd22) and WT worms on E. coli OP50. Lifespan assay of L4 larval stage wild-type worms and atf-7(qd22) mutant animals on E. coli OP50. Both strains carry the agIs219 transgene. Replicate data can be seen in Figure S16. (0.13 MB PDF) [file pgen.1000892.s001.pdf]

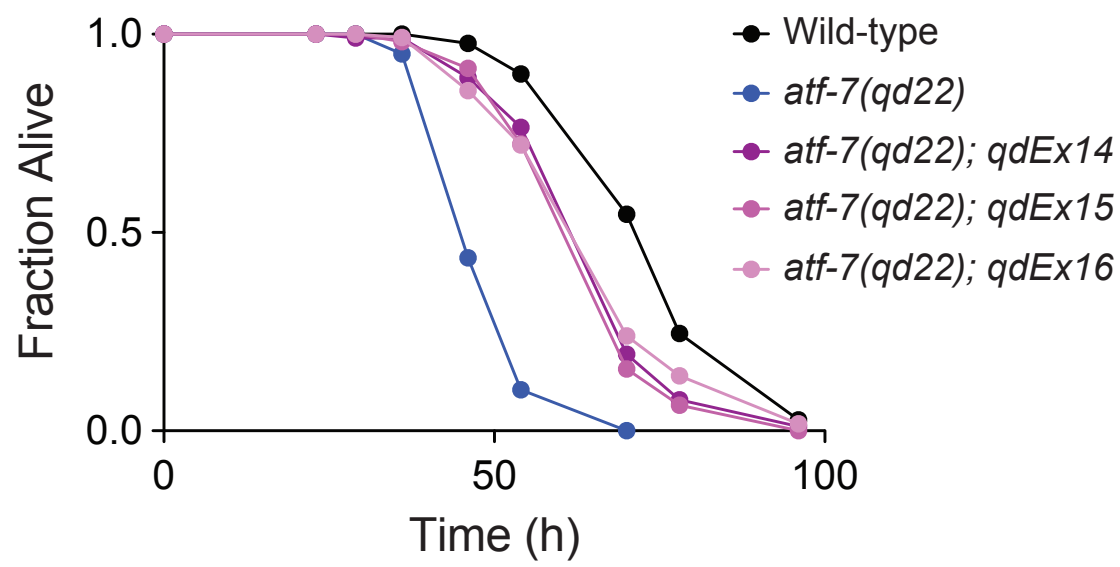

Supplement: Figure S2 — Rescue of atf-7(qd22) Esp phenotype. Pathogenesis assay of L4 larval stage wild-type worms, atf-7(qd22) mutant animals, and three independent transgenic lines (qdEx14, qdEx15, and qdEx16) of atf-7(qd22) mutant animals carrying fosmid 25cA04. All strains carry the agIs219 transgene. The difference in susceptibility between atf-7(qd22) mutant animals and each transgenic line carrying fosmid 25cA04 is significant (p<0.0001). (0.13 MB PDF) [file pgen.1000892.s002.pdf]

A

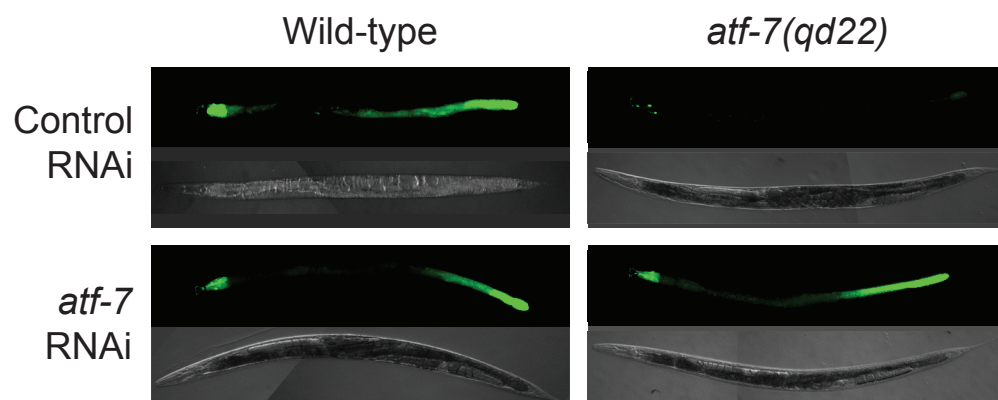

B

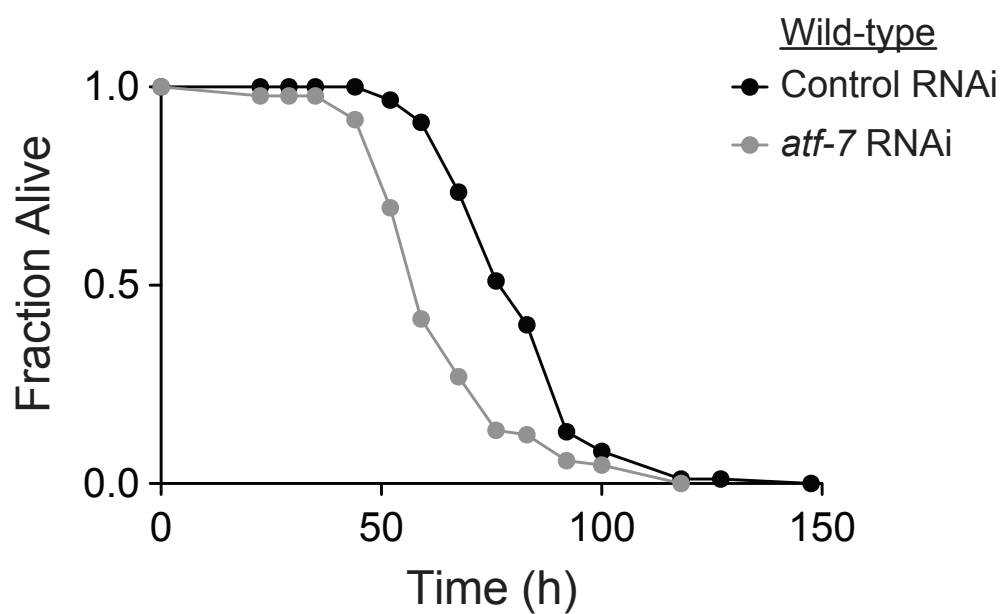

C

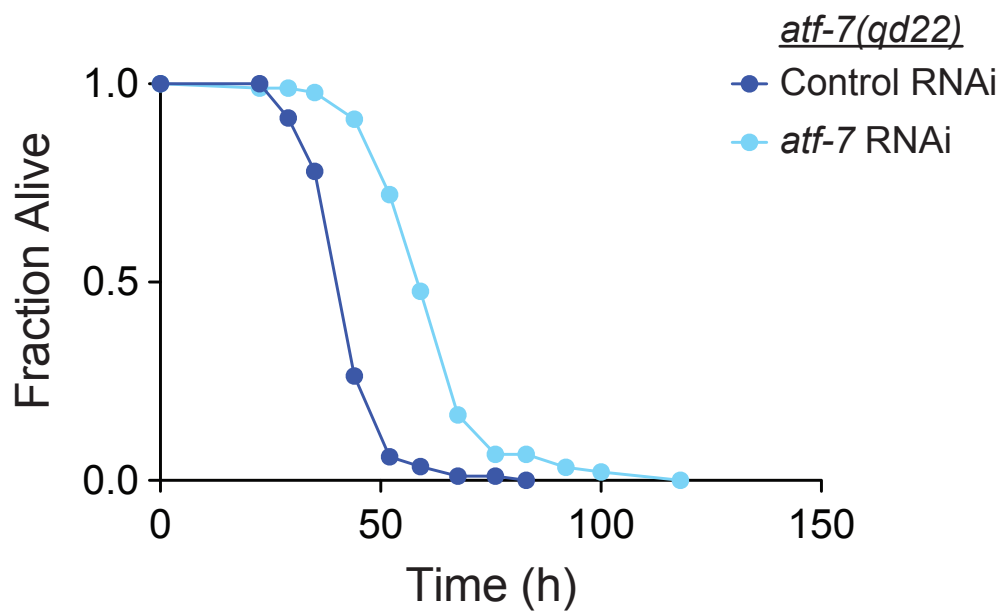

Supplement: Figure S3 — atf-7(qd22) is a gain-of-function allele. (A) Fluorescence microscopy images of GFP expression from the agIs219 transgene in wild-type worms and atf-7(qd22) mutant worms each exposed to both control RNAi and RNAi of atf-7. (B) Pathogenesis assay comparing the effects of control RNAi and RNAi of atf-7 on survival of wild-type worms on P. aeruginosa PA14. The difference in susceptibility between wild-type worms treated with control RNAi and atf-7 RNAi is significant (p<0.0001). Replicate data can be seen in Figure S11. (C) Pathogenesis assay comparing the effects of control RNAi and RNAi of atf-7 on survival of atf-7(qd22) mutant animals on P. aeruginosa PA14. The difference in susceptibility between atf-7(qd22) mutant animals treated with control RNAi and atf-7 RNAi is significant (p<0.0001). Replicate data can be seen in Figure S11. (0.30 MB PDF) [file pgen.1000892.s003.pdf]

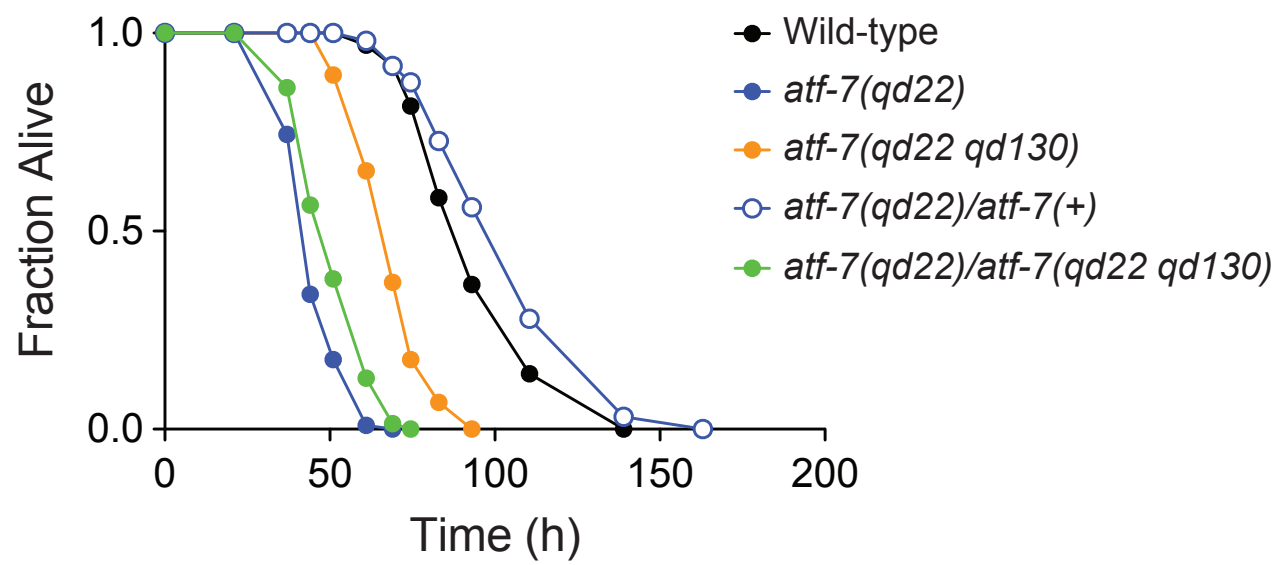

Supplement: Figure S4 — atf-7(qd22) confers a recessive Esp phenotype. Pathogenesis assay of L4 larval stage wild-type worms; atf-7(qd22) and atf-7(qd22 qd130) mutant animals; and atf-7(qd22)/atf-7(+) and atf-7(qd22)/atf-7(qd22 qd130) trans-heterozygotes, on P. aeruginosa PA14. All strains carry the agIs219 transgene. The differences in susceptibility between atf-7(qd22) mutant animals and atf-7(qd22)/atf-7(+) trans-heterozygotes, and atf-7(qd22 qd130) mutant animals and atf-7(qd22)/atf-7(qd22 qd130) trans-heterozygotes are significant (p<0.0001 for each comparison). Replicate data can be seen in Figure S12. (0.13 MB PDF) [file pgen.1000892.s004.pdf]

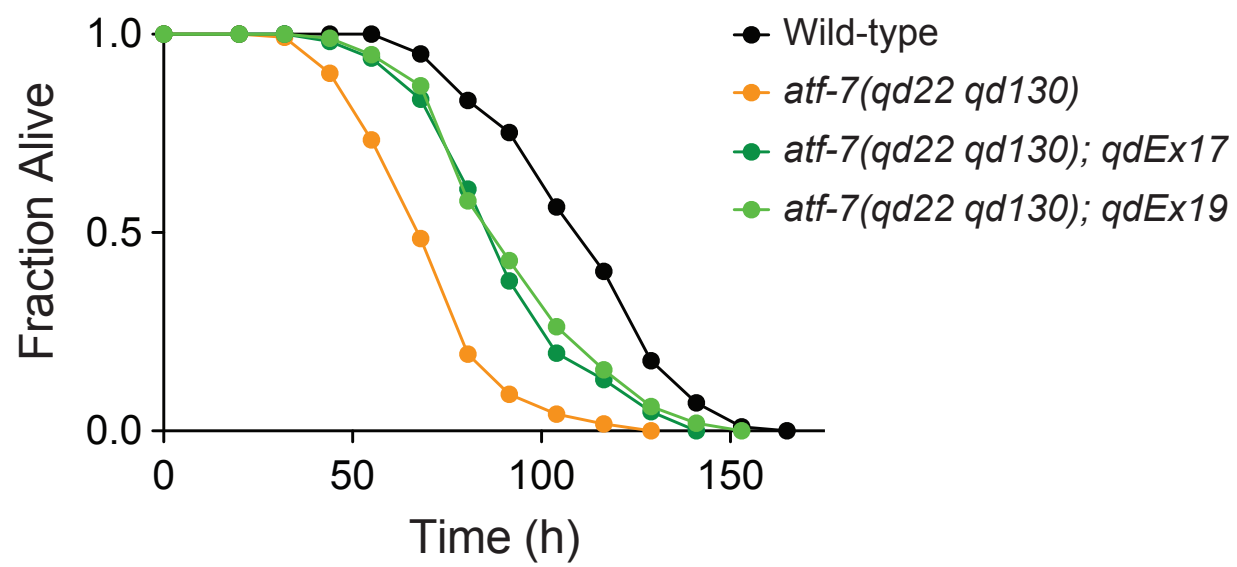

Supplement: Figure S5 — Expression of atf-7::GFP rescues the atf-7(qd22 qd130) Esp phenotype. Pathogenesis assay of wild-type worms, atf-7(qd22 qd130) mutant animals, and two independent transgenic lines (qdEx17 and qdEx19) of atf-7(qd22 qd130) mutant animals carrying the atf-7::GFP construct under the control of the endogenous atf-7 genomic promoter and 3′- untranslated region, on P. aeruginosa PA14. All strains carry the agIs219 transgene. The difference in susceptibility between atf-7(qd22 qd130) mutant animals and each transgenic line carrying the atf-7::GFP transgene is significant (p<0.0001 for each comparison). (0.13 MB PDF) [file pgen.1000892.s005.pdf]

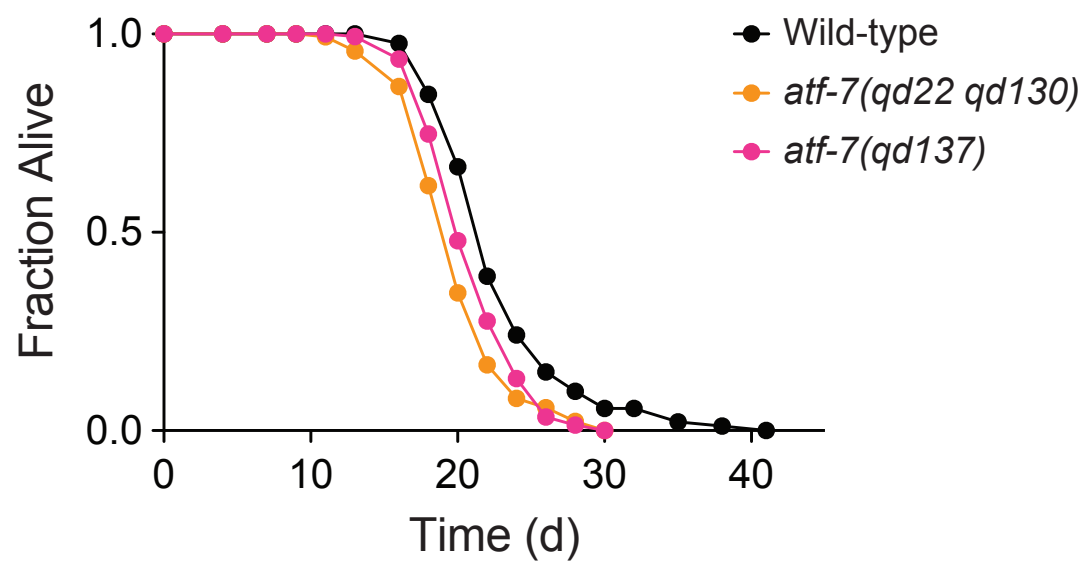

Supplement: Figure S6 — Lifespan of atf-7 loss-of-function mutants and WT worms on E. coli OP50. Lifespan assay of L4 larval stage wild-type worms, atf-7(qd22 qd130) and atf-7(qd137) mutant animals on E. coli OP50. All strains carry the agIs219 transgene. Replicate data can be seen in Figure S16. (0.13 MB PDF) [file pgen.1000892.s006.pdf]

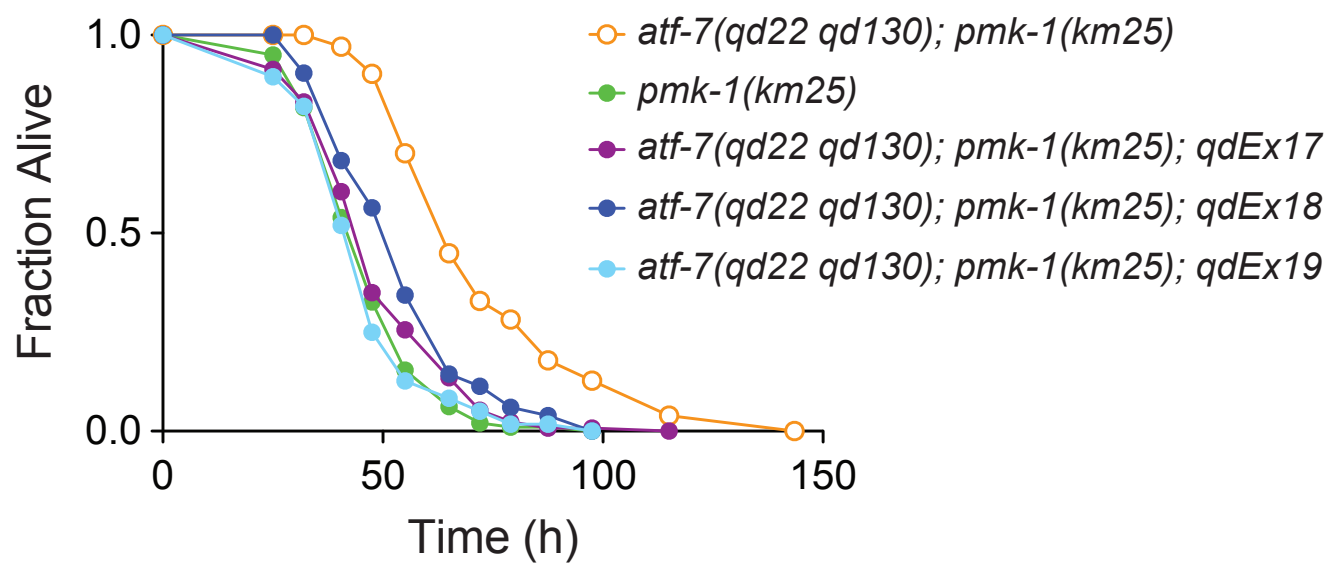

Supplement: Figure S7 — Expression of atf-7::GFP rescues the atf-7(qd22 qd130) suppression of pmk-1(km25). Pathogenesis assay of pmk-1(km25) mutant animals and atf-7(qd22 qd130); pmk-1(km25) double mutant animals, along with three independent transgenic lines (qdEx17, qdEx18, and qdEx19) of atf-7(qd22 qd130); pmk-1(km25) double mutant animals carrying the atf-7::GFP construct under the control of the endogenous atf-7 genomic promoter and 3′-untranslated region, on P. aeruginosa PA14. All strains carry the agIs219 transgene. The difference in susceptibility between atf-7(qd22 qd130); pmk-1(km25) double mutant animals and each transgenic line carrying the atf-7::GFP transgene is significant (p<0.0001 for each comparison). (0.13 MB PDF) [file pgen.1000892.s007.pdf]

|            |                                                                                                                  |   |   |   |
|------------|------------------------------------------------------------------------------------------------------------------|---|---|---|
| T7-ATF-7   | +                                                                                                                | + | + | + |
| HA-PMK-1   | -                                                                                                                | + | + | + |
| FLAG-SEK-1 | -                                                                                                                | - | + | + |
| PPase      | -                                                                                                                | - | - | + |
| IP:T7      | <div> <div>α T7</div> 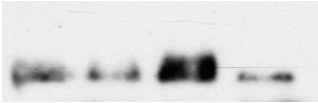 </div>   |   |   |   |
| WCE        | <div> <div>α HA</div> 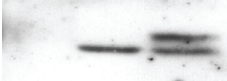 </div>   |   |   |   |
|            | <div> <div>α FLAG</div> 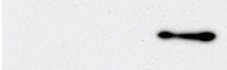 </div> |   |   |   |

Supplement: Figure S8 — PMK-1 phosphorylation of ATF-7 is sensitive to phosphatase. Cos7 cells were transfected with T7-ATF-7, HA-PMK-1, and FLAG-SEK-1 as indicated. ATF-7 was immunoprecipitated with anti-T7, treated with phosphatase where indicated, and immunoblotted with anti-T7 (top). Whole cell extracts were immunoblotted with antibodies that recognize HA (middle) and FLAG (bottom). (0.20 MB PDF) [file pgen.1000892.s008.pdf]

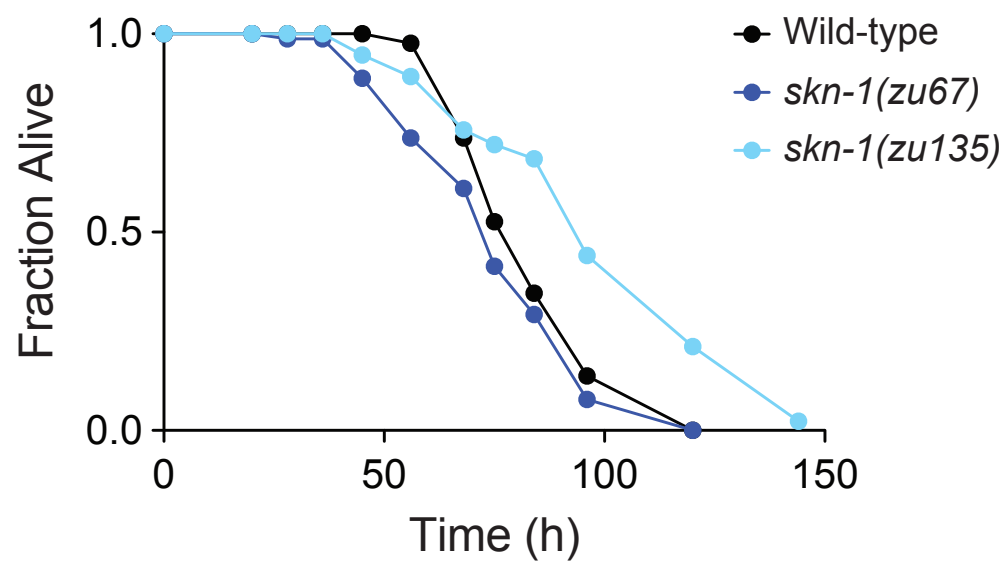

Supplement: Figure S9 — skn-1 mutants do not exhibit an Esp phenotype. Pathogenesis assay of L4 larval stage wild-type worms, skn-1(zu67) and skn-1(zu135) mutant animals on P. aeruginosa PA14. (0.13 MB PDF) [file pgen.1000892.s009.pdf]
