# Supplementary material for: Phosphorylation of the Conserved Transcription Factor ATF-7 by PMK-1 p38 MAPK Regulates Innate Immunity in Caenorhabditis elegans
Source: PLoS Genet. 2010 Apr 1;6(4):e1000892. doi: 10.1371/journal.pgen.1000892 (PMC2848548; doi:10.1371/journal.pgen.1000892)

Experiment #1

Genotype	Mean LT_{50} (h)	LT_{50} S.D. (h)	Sample Size (n)
Wild-type	103.6	5.7	94
atf-7(qd22)	43.2	4.2	112
atf-7(qd22 qd130)	58.6	4.6	119

Experiment #2

Genotype	Mean LT_{50} (h)	LT_{50} S.D. (h)	Sample Size (n)
Wild-type	110.1	8.2	93
atf-7(qd22)	44.7	1.5	101
atf-7(qd22 qd130)	58.2	3.7	79

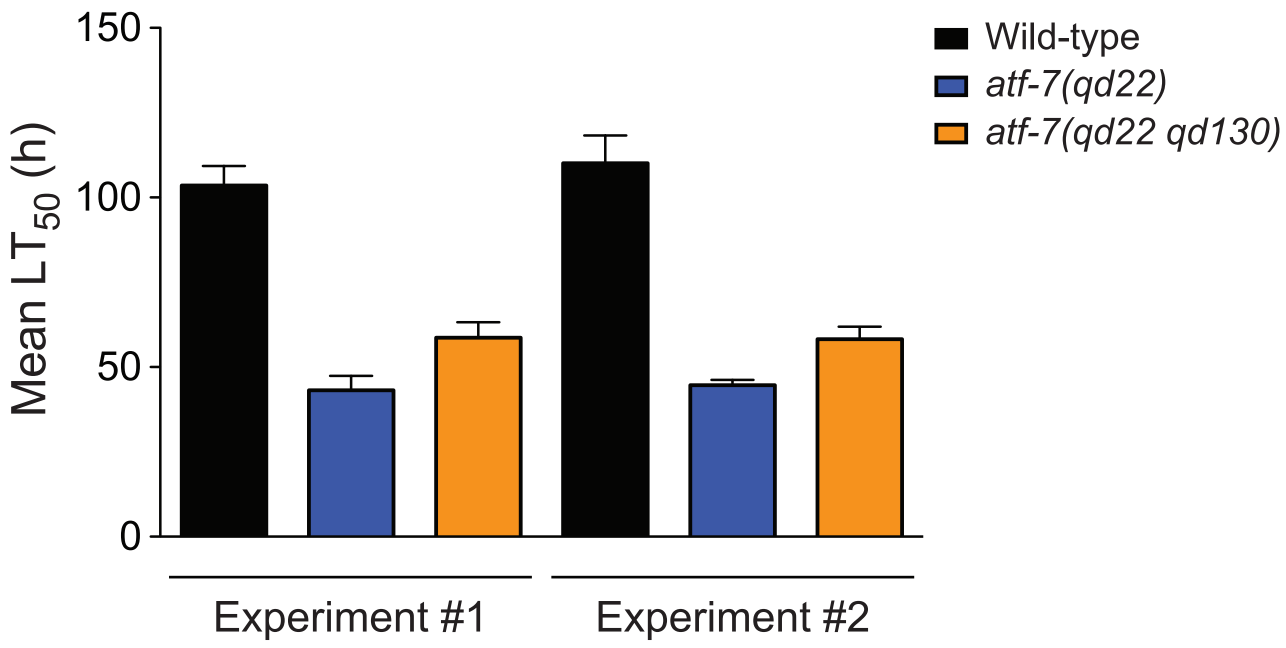

Supplement: Figure S10 — Replicate of pathogenesis assay shown in Figure 1B. Chart and bar graphs showing the LT50 means, LT50 standard deviations (S.D.), and sample sizes from two independent P. aeruginosa pathogenesis assays with wild-type worms, atf-7(qd22) and atf-7(qd22 qd130) mutant animals. (0.18 MB PDF) [file pgen.1000892.s010.pdf]
