# Supplementary material for: Phosphorylation of the Conserved Transcription Factor ATF-7 by PMK-1 p38 MAPK Regulates Innate Immunity in Caenorhabditis elegans
Source: PLoS Genet. 2010 Apr 1;6(4):e1000892. doi: 10.1371/journal.pgen.1000892 (PMC2848548; doi:10.1371/journal.pgen.1000892)

Experiment #1

Genotype	Treatment	Mean LT_{50} (h)	LT_{50} S.D. (h)	Sample Size (n)
Wild-type	Control	77.1	2.8	74
	atf-7 RNAi	57.9	6.9	69
atf-7(qd22)	Control	39.8	1.6	83
	atf-7 RNAi	57.8	2.5	90

Experiment #2

Genotype	Treatment	Mean LT_{50} (h)	LT_{50} S.D. (h)	Sample Size (n)
Wild-type	Control	54.2	3.3	69
	atf-7 RNAi	42.0	2.6	79
atf-7(qd22)	Control	36.1	3.4	86
	atf-7 RNAi	46.7	3.8	85

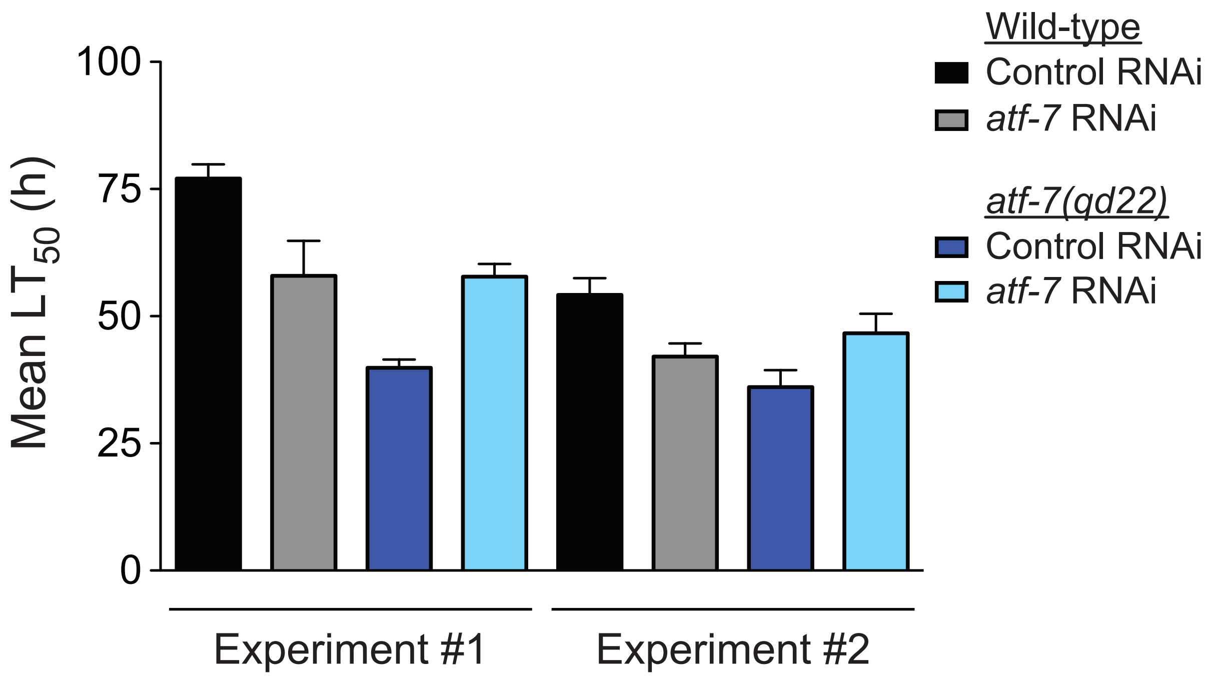

Supplement: Figure S11 — Replicate of pathogenesis assay shown in Figure S3B and S3C. Chart and bar graphs showing the LT50 means, LT50 standard deviations (S.D.), and sample sizes from two independent P. aeruginosa pathogenesis assays with wild-type worms treated with control RNAi and atf-7 RNAi, and atf-7(qd22) mutant animals treated with control RNAi and atf-7 RNAi. (0.19 MB PDF) [file pgen.1000892.s011.pdf]
