# Supplementary material for: Phosphorylation of the Conserved Transcription Factor ATF-7 by PMK-1 p38 MAPK Regulates Innate Immunity in Caenorhabditis elegans
Source: PLoS Genet. 2010 Apr 1;6(4):e1000892. doi: 10.1371/journal.pgen.1000892 (PMC2848548; doi:10.1371/journal.pgen.1000892)

Experiment #1

Genotype	Mean LT_{50} (h)	LT_{50} S.D. (h)	Sample Size (n)
Wild-type	87.6	9.5	95
atf-7(qd22)	41.6	2.4	109
atf-7(qd22 qd130)	65.2	2.0	103
atf-7(qd22)/atf-7(+)	96.6	3.3	96
atf-7(qd22 qd130)/atf-7(+)	102.7	2.3	86
atf-7(qd22)/atf-7(qd22 qd130)	47.1	1.1	71

Experiment #2

Genotype	Mean LT_{50} (h)	LT_{50} S.D. (h)	Sample Size (n)
Wild-type	88.9	2.8	79
atf-7(qd22)	43.4	3.2	105
atf-7(qd22 qd130)	52.0	1.8	89
atf-7(qd22)/atf-7(+)	83.9	3.6	103
atf-7(qd22 qd130)/atf-7(+)	91.6	8.2	96
atf-7(qd22)/atf-7(qd22 qd130)	45.2	1.3	106

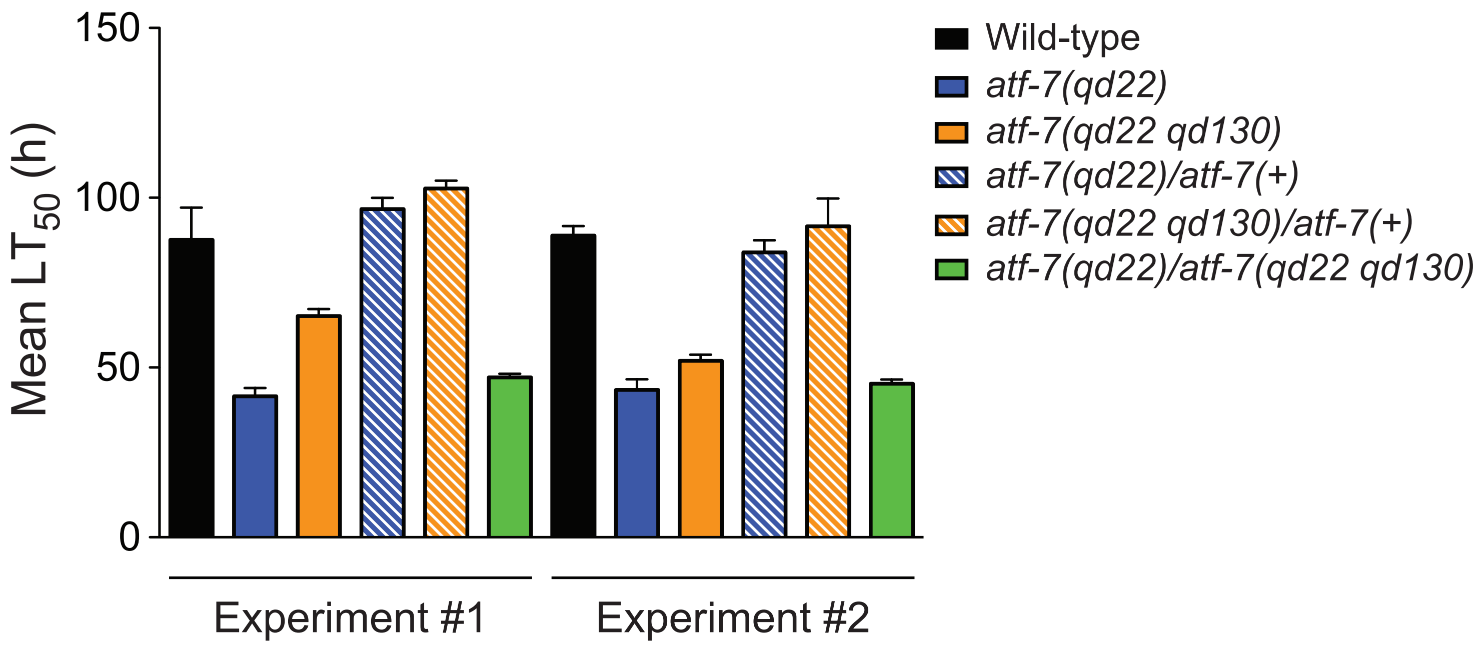

Supplement: Figure S12 — Replicate of pathogenesis assay shown in Figure S4. Chart and bar graphs showing the LT50 means, LT50 standard deviations (S.D.), and sample sizes from two independent P. aeruginosa pathogenesis assays with wild-type worms; atf-7(qd22) and atf-7(qd22 q130) mutant animals; and atf-7(qd22)/atf-7(+), atf-7(qd22 qd130)/atf-7(+), and atf-7(qd22)/atf-7(qd22 qd130) trans-heterozygotes. (0.20 MB PDF) [file pgen.1000892.s012.pdf]
