# Supplementary material for: Phosphorylation of the Conserved Transcription Factor ATF-7 by PMK-1 p38 MAPK Regulates Innate Immunity in Caenorhabditis elegans
Source: PLoS Genet. 2010 Apr 1;6(4):e1000892. doi: 10.1371/journal.pgen.1000892 (PMC2848548; doi:10.1371/journal.pgen.1000892)

Experiment #1

Genotype	Mean LT_{50} (h)	LT_{50} S.D. (h)	Sample Size (n)
Wild-type	104.7	4.2	95
atf-7(qd22 qd130)	66.0	9.0	111
atf-7(qd137)	60.3	4.3	113
atf-7(qd22 qd130)/atf-7(+)	114.9	8.0	116
atf-7(qd137)/atf-7(+)	122.8	12.8	103
atf-7(qd22 qd130)/atf-7(qd137)	62.9	2.2	119

Experiment #2

Genotype	Mean LT_{50} (h)	LT_{50} S.D. (h)	Sample Size (n)
Wild-type	104.5	4.2	116
atf-7(qd22 qd130)	61.4	1.3	126
atf-7(qd137)	59.8	1.6	119
atf-7(qd22 qd130)/atf-7(+)	126.6	12.1	118
atf-7(qd137)/atf-7(+)	105.8	3.9	129
atf-7(qd22 qd130)/atf-7(qd137)	60.3	1.5	134

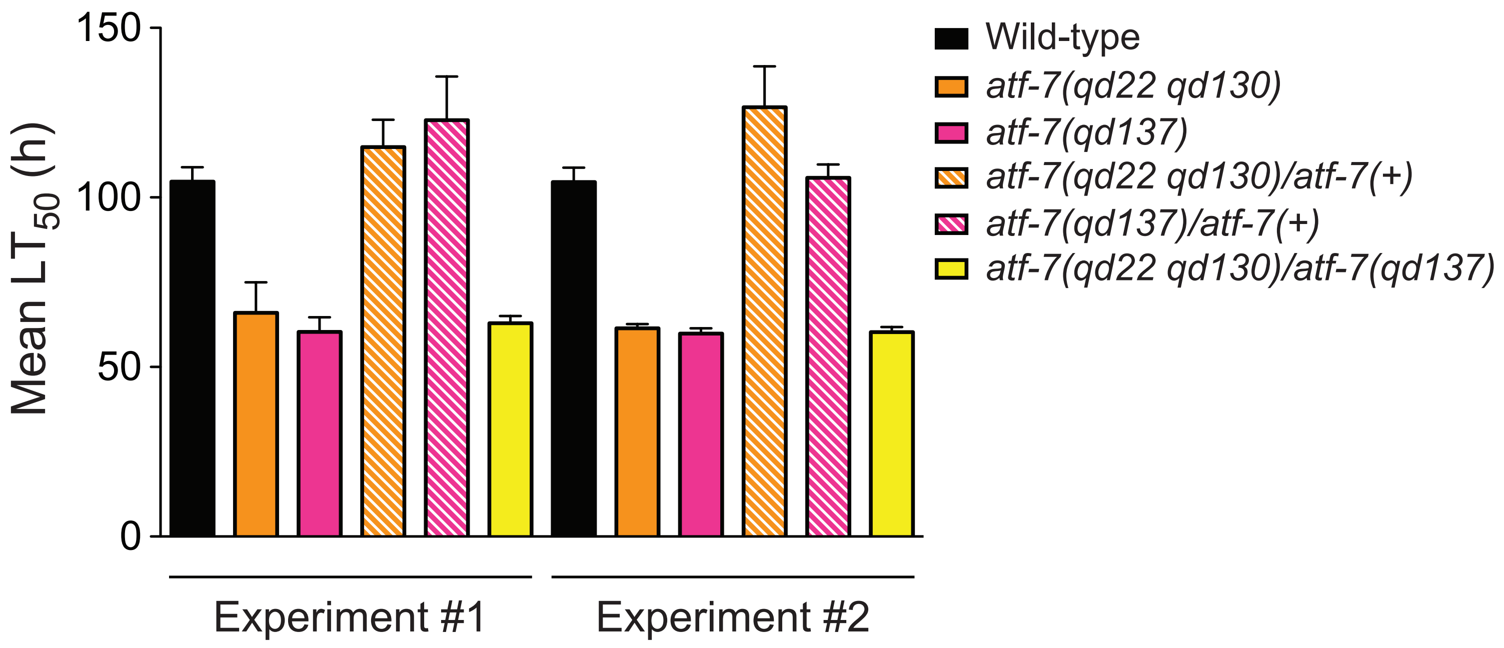

Supplement: Figure S13 — Replicate of pathogenesis assay shown in Figure 1D. Chart and bar graphs showing the LT50 means, LT50 standard deviations (S.D.), and sample sizes from two independent P. aeruginosa pathogenesis assays with wild-type worms; atf-7(qd22 q130) and atf-7(qd137) mutant animals; and atf-7(qd22 qd130)/atf-7(+), atf-7(qd137)/atf-7(+), and atf-7(qd22 qd130)/atf-7(qd137) trans-heterozygotes. (0.20 MB PDF) [file pgen.1000892.s013.pdf]
