# Supplementary material for: Phosphorylation of the Conserved Transcription Factor ATF-7 by PMK-1 p38 MAPK Regulates Innate Immunity in Caenorhabditis elegans
Source: PLoS Genet. 2010 Apr 1;6(4):e1000892. doi: 10.1371/journal.pgen.1000892 (PMC2848548; doi:10.1371/journal.pgen.1000892)

Experiment #1

Genotype	Mean LT_{50} (h)	LT_{50} S.D. (h)	Sample Size (n)
Wild-type	106.2	6.1	96
atf-7(qd22 qd130)	67.5	1.5	114
atf-7(qd22 qd130); pmk-1(km25)	59.2	1.5	106
pmk-1(km25)	41.3	1.1	106

Experiment #2

Genotype	Mean LT_{50} (h)	LT_{50} S.D. (h)	Sample Size (n)
Wild-type	103.6	5.7	94
atf-7(qd22 qd130)	58.6	4.6	119
atf-7(qd22 qd130); pmk-1(km25)	52.8	6.7	111
pmk-1(km25)	42.4	1.5	120

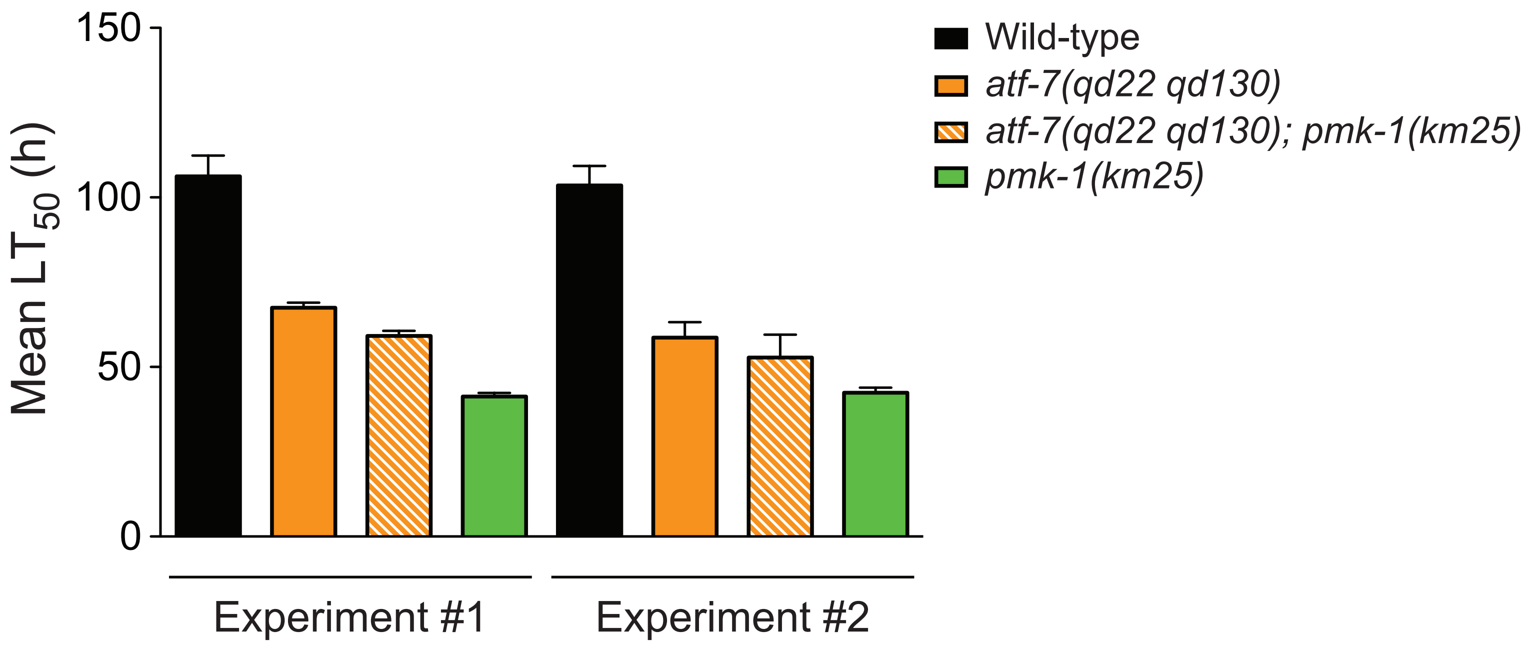

Supplement: Figure S14 — Replicate of pathogenesis assay shown in Figure 3B. Chart and bar graphs showing the LT50 means, LT50 standard deviations (S.D.), and sample sizes from two independent P. aeruginosa pathogenesis assays with wild-type worms; atf-7(qd22 q130) and pmk-1(km25) mutant animals; and atf-7(qd22 qd130); pmk-1(km25) double mutant animals. (0.19 MB PDF) [file pgen.1000892.s014.pdf]
