# Supplementary material for: Phosphorylation of the Conserved Transcription Factor ATF-7 by PMK-1 p38 MAPK Regulates Innate Immunity in Caenorhabditis elegans
Source: PLoS Genet. 2010 Apr 1;6(4):e1000892. doi: 10.1371/journal.pgen.1000892 (PMC2848548; doi:10.1371/journal.pgen.1000892)

Experiment #1

Genotype	Mean LT_{50} (h)	LT_{50} S.D. (h)	Sample Size (n)
Wild-type	98.7	5.9	77
atf-7(qd22 qd130)	57.7	0.7	95
atf-7(qd22 qd130); sek-1(km4)	53.9	3.3	97
sek-1(km4)	33.1	1.9	125

Experiment #2

Genotype	Mean LT_{50} (h)	LT_{50} S.D. (h)	Sample Size (n)
Wild-type	95.4	8.8	74
atf-7(qd22 qd130)	54.6	1.9	85
atf-7(qd22 qd130); sek-1(km4)	58.4	2.3	80
sek-1(km4)	33.8	1.1	111

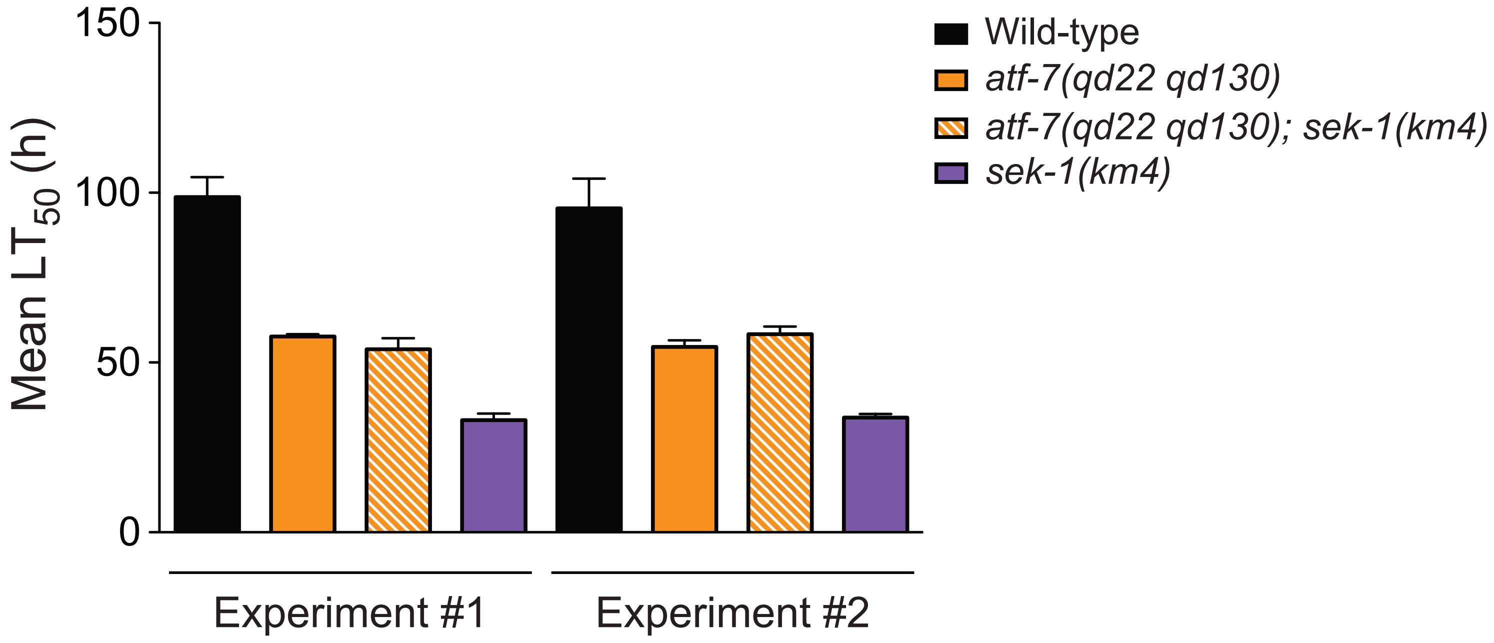

Supplement: Figure S15 — Replicate of pathogenesis assay shown in Figure 3D. Chart and bar graphs showing the LT50 means, LT50 standard deviations (S.D.), and sample sizes from two independent P. aeruginosa pathogenesis assays with wild-type worms; atf-7(qd22 q130) and sek-1(km4) mutant animals; and atf-7(qd22 qd130); sek-1(km4) double mutant animals. (0.19 MB PDF) [file pgen.1000892.s015.pdf]
