# Supplementary material for: Phosphorylation of the Conserved Transcription Factor ATF-7 by PMK-1 p38 MAPK Regulates Innate Immunity in Caenorhabditis elegans
Source: PLoS Genet. 2010 Apr 1;6(4):e1000892. doi: 10.1371/journal.pgen.1000892 (PMC2848548; doi:10.1371/journal.pgen.1000892)

Experiment #1

Genotype	Mean LT_{50} (d)	LT_{50} S.D. (d)	Sample Size (n)
Wild-type	21.1	0.9	67
atf-7(qd22)	19.1	0.5	152
atf-7(qd22 qd130)	18.9	1.1	124
atf-7(qd137)	20.0	1.1	144

Experiment #2

Genotype	Mean LT_{50} (d)	LT_{50} S.D. (d)	Sample Size (n)
Wild-type	22.3	1.5	45
atf-7(qd22)	19.4	0.9	107
atf-7(qd22 qd130)	18.5	1.4	97
atf-7(qd137)	17.4	1.5	158

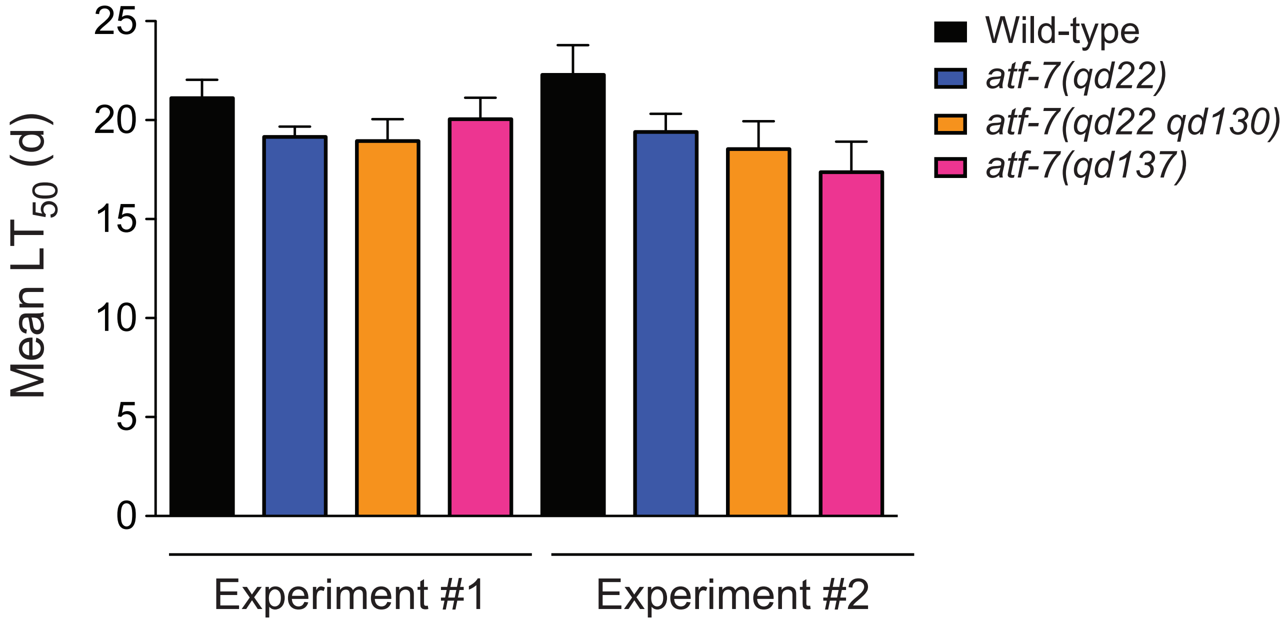

Supplement: Figure S16 — Replicate of lifespan assay shown in Figure S1 and Figure S6. Chart and bar graphs showing the LT50 means, LT50 standard deviations (S.D.), and sample sizes from two independent lifespan assays with wild-type worms, atf-7(qd22), atf-7(qd22 q130), and atf-7(qd137) mutant animals. (0.18 MB PDF) [file pgen.1000892.s016.pdf]
