# Supplementary material for: Phosphorylation of the Conserved Transcription Factor ATF-7 by PMK-1 p38 MAPK Regulates Innate Immunity in Caenorhabditis elegans
Source: PLoS Genet. 2010 Apr 1;6(4):e1000892. doi: 10.1371/journal.pgen.1000892 (PMC2848548; doi:10.1371/journal.pgen.1000892)
Supplement: Table S1 — Sample sizes for each pathogenesis, arsenite stress, and lifespan assay. Chart showing the sample sizes for each pathogenesis, arsenite stress, and lifespan assay presented in this work. Sample size does not include censored worms. (0.16 MB PDF) [file pgen.1000892.s017.pdf]

Figure	Genotype	Sample Size (n)
1B	Wild-type	94
	atf-7(qd22)	112
	atf-7(qd22 qd130)	119
1D	Wild-type	95
	atf-7(qd22 qd130)	111
	atf-7(qd137)	113
	atf-7(qd22 qd130)/atf-7(+)	116
	atf-7(qd137)/atf-7(+)	103
	atf-7(qd22 qd130)/atf-7(qd137)	119
3B	Wild-type	96
	atf-7(qd22 qd130)	114
	atf-7(qd22 qd130); pmk-1(km25)	106
	pmk-1(km25)	106
3D	Wild-type	77
	atf-7(qd22 qd130)	95
	atf-7(qd22 qd130); sek-1(km4)	97
	sek-1(km4)	125
7A	Wild-type	121
	atf-7(qd22 qd130)	103
	atf-7(qd22 qd130); pmk-1(km25)	115
	pmk-1(km25)	115
7B	Wild-type	73
	atf-7(qd22 qd130)	73
	atf-7(qd22 qd130); pmk-1(km25)	92
	pmk-1(km25)	77
8	Wild-type	77
	atf-7(qd22 qd130)	68
	atf-7(qd22 qd130); pmk-1(km25)	67
	pmk-1(km25)	82
	atf-7(qd22 qd130); sek-1(km4)	77
	sek-1(km4)	74
S1	Wild-type	67
	atf-7(qd22)	152
S2	Wild-type	68
	atf-7(qd22)	81
	atf-7(qd22); qdEx14	103
	atf-7(qd22); qdEx15	93
	atf-7(qd22); qdEx16	100
S3B	[Wild-type] Control RNAi	74
	[Wild-type] *atf-7* RNAi	69
S3C	[*atf-7(qd22)*] Control RNAi	83
	[*atf-7(qd22)*] *atf-7* RNAi	90
S4	Wild-type	95
	atf-7(qd22)	109
	atf-7(qd22)/atf-7(+)	96
	atf-7(qd22)/atf-7(qd22 qd130)	71
S5	Wild-type	94
	atf-7(qd22 qd130)	119
	atf-7(qd22 qd130); qdEx17	114
	atf-7(qd22 qd130); qdEx19	99
S6	Wild-type	67
	atf-7(qd22 qd130)	124
	atf-7(qd137)	144
S7	*atf-7(qd22 qd130); pmk-1(km25)*	100
	pmk-1(km25)	98
	atf-7(qd22 qd130); pmk-1(km25); qdEx17	78
	atf-7(qd22 qd130); pmk-1(km25); qdEx18	62
	atf-7(qd22 qd130); pmk-1(km25); qdEx19	66
